# The mechanistic link between selective vulnerability of the locus coeruleus and neurodegeneration in Alzheimer’s disease

**DOI:** 10.1007/s00401-020-02248-1

**Published:** 2021-01-11

**Authors:** Billie J. Matchett, Lea T. Grinberg, Panos Theofilas, Melissa E. Murray

**Affiliations:** 1 Neuropathology Laboratory, Department of Neuroscience, Mayo Clinic, 4500 San Pablo Road, Jacksonville, FL 32224 USA; 2 Memory and Aging Center, Department of Neurology, University of California, 675 Nelson Rising Lane, San Francisco, CA 94158 USA

**Keywords:** Locus coeruleus, Alzheimer’s disease, Tau, Norepinephrine, Selective vulnerability

## Abstract

Alzheimer’s disease (AD) is neuropathologically characterized by the intracellular accumulation of hyperphosphorylated tau and the extracellular deposition of amyloid-β plaques, which affect certain brain regions in a progressive manner. The locus coeruleus (LC), a small nucleus in the pons of the brainstem, is widely recognized as one of the earliest sites of neurofibrillary tangle formation in AD. Patients with AD exhibit significant neuronal loss in the LC, resulting in a marked reduction of its size and function. The LC, which vastly innervates several regions of the brain, is the primary source of the neurotransmitter norepinephrine (NE) in the central nervous system. Considering that NE is a major modulator of behavior, contributing to neuroprotection and suppression of neuroinflammation, degeneration of the LC in AD and the ultimate dysregulation of the LC–NE system has detrimental effects in the brain. In this review, we detail the neuroanatomy and function of the LC, its essential role in neuroprotection, and how this is dysregulated in AD. We discuss AD-related neuropathologic changes in the LC and mechanisms by which LC neurons are selectively vulnerable to insult. Further, we elucidate the neurotoxic effects of LC de-innervation both locally and at projection sites, and how this augments disease pathology, progression and severity. We summarize how preservation of the LC–NE system could be used in the treatment of AD and other neurodegenerative diseases affected by LC degeneration.

## Introduction

The locus coeruleus (LC) is a small brainstem pontine nucleus of neuromelanin-containing neurons, widely recognized as the primary source of the mono-aminergic neurotransmitter norepinephrine (NE). NE plays an important role in modulating many behavioral functions, including attention, mood, motivation, stress and arousal [96, 134]. Furthermore, NE influences blood flow, heart rate, sleep and waking patterns [80, 91], and regulates neuroinflammation and neuronal survival [94]. Thus, the noradrenergic system was established as one of the most pivotal neuromodulators in the brain.

Current research supports that dysregulation of the noradrenergic system is a significant player in the development of psychiatric and neurodegenerative disorders, including Alzheimer’s disease (AD), Parkinson’s disease, Lewy body dementia, and frontotemporal lobar degeneration with tau. Analyses of AD brains from progressive stages of the disease have reported significant neuronal and volume loss in the LC [64, 154]. Previous studies have suggested that LC degeneration is age related [50, 85, 93, 157, 164]. However, it is of note that some of these findings were based on a small cohort (*n* = 5–13) [50, 85] and did not exclude cases with neuropathology elsewhere. Intriguingly, recent unbiased stereological approaches indicated that there are no significant associations between normal aging and changes in the LC [16, 106, 116, 154], suggesting a disease-specific phenomenon.

AD is neuropathologically characterized by extracellular amyloid-β (Aβ) plaque deposits and abnormal accumulation of intracellular hyperphosphorylated tau (p-tau) that form neurofibrillary tangles (NFTs) [103]. In 1991, Braak and Braak defined the six-stage pattern of NFT deposition across the course of AD neuropathologic progression [10]. In 2011, Braak et al*.* revised the standardized staging scheme to include pretangle stages a–c, which denote the accumulation of subcortical p-tau in the LC that occurs before any cortical tau pathology [12]. This observation situates the LC, together with other interconnected neuromodulatory subcortical structures [143, 153], as one of the first structures to accumulate AD-tau as neuronal inclusions [12], a pathology that ultimately results in neuronal death. The subsequent loss of LC neurons, which occurs with a topographical gradient, is associated with increased Aβ plaque deposition and NFT load in the cortices [8]. Dysfunction of the LC noradrenergic system was also found to be associated with the onset of memory dysfunction and cognitive impairment in AD [55]. Interestingly, LC degeneration correlated better with AD onset and duration than degeneration of the cholinergic nucleus basalis of Meynert (nbM) in the basal forebrain [100, 169], which is also highly vulnerable to tau pathology in AD, indicating that noradrenergic deficits in AD play a major role in disease progression. These findings show that the connection between loss of LC noradrenergic innervation and subsequent onset of neurodegeneration in AD, as well as the detection and treatment of noradrenergic deficits in patients, are important disease aspects that require further research and understanding.

The LC has a unique therapeutic value in AD as it is one of the earliest subcortical regions affected by tau lesions, and prevention of neuropathologic changes in this nucleus could prevent the spread of irreversible changes in the brain [61, 67]. With this in mind, this review will summarize the neuroanatomy of the LC and its function within the noradrenergic system, stating the mechanisms by which NE exerts its responsibility as a key neuromodulator, as well as its role in neuroprotection. We will discuss which AD-related neuropathologic changes are observed in the LC, how the LC–NE system’s dysregulation leads to AD neuropathology, onset and progression, and possible therapeutic interventions to prevent or lessen LC degeneration and NE deficits in AD.

## Neuroanatomy and function of the locus coeruleus

The LC is a “tube-like” collection of noradrenergic neurons located in the dorsolateral pontine tegmentum, beginning rostrally at the inferior colliculus level and continuing caudally to the lateral face of the fourth ventricle [50, 68]. The LC forms part of the isodendritic core, a group of interconnected and phylogenetically conserved subcortical nuclei [125, 153]. This group also includes the dorsal raphe nucleus, substantia nigra in the midbrain, and nbM in the forebrain [125, 153]. The isodendritic core network plays an important role in neuromodulation by regulating behavior and homeostasis through aminergic and cholinergic projections to the cortices [153].

**Fig. 1:**
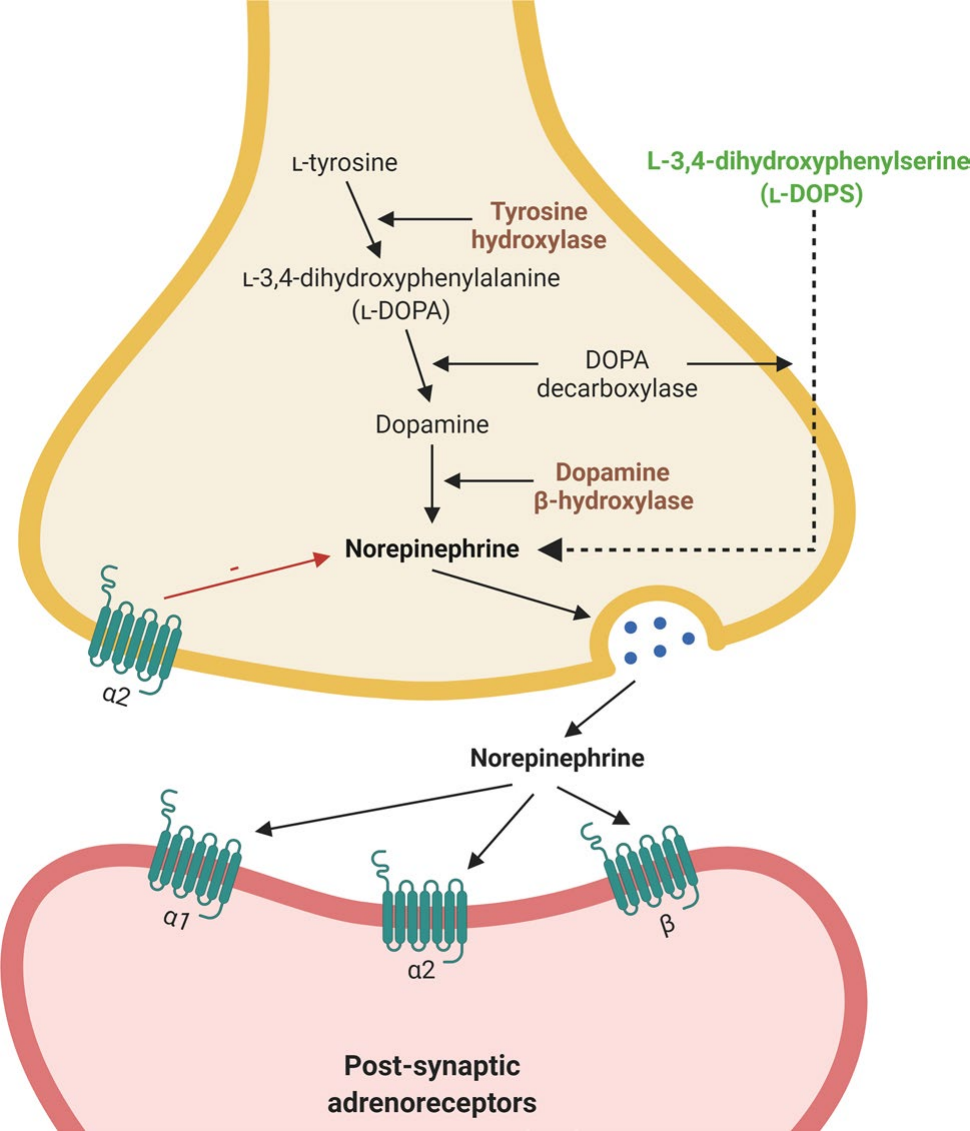
Norepinephrine biosynthetic and signaling pathways in locus coeruleus neurons. l-tyrosine is converted into norepinephrine by a series of enzymatic reactions [70, 113]. Antibody immunoreactivity to key enzymes in the norepinephrine pathway is commonly used as markers of norepinephrine neurons [24]. norepinephrine is released at the synapse, where it binds to G-coupled α- and β-adrenoreceptors to exert its neuromodulatory effects. Agents such as l-3,4-dihydroxyphenylserine [149] can bypass the pathway and directly increase levels of norepinephrine and, thus, could be used as a potential therapy to compensate for norepinephrine loss following locus coeruleus de-innervation. Created with BioRender.com

Unbiased stereological estimates of the total LC cell population showed an average of 98,000 neurons, constituting an overall volume of approximately 13 mm^3^ [154]. LC neurons are identifiable by their neuromelanin pigment, as well as immunolabeling with dopamine β-hydroxylase, the enzyme that converts dopamine to NE [138, 146] or tyrosine hydroxylase, the enzyme that catalyzes the rate-limiting step in NE biosynthesis [121] (Fig. 1). Using such methods, several studies have identified distinct cellular heterogeneity within the LC, characterizing two classes of medium-sized neurons; the large multipolar cells (~ 35 μm) and smaller fusiform cells (~ 20 μm) [138, 145]. Their distinct cellular morphologies could indicate differences in their characteristics and function. Though both cell types are dispersed throughout the LC, the small fusiform cells dominate the densely packed rostral portion, indicating a cytoarchitectonic bias [145]. In addition, recent studies have indicated phenotypic variability among subsets of LC neurons [17, 124, 165]. Hippocampal and prefrontal-innervating LC neurons differ in their physiological response to the α_2_-adrenoreceptor agonist clonidine, suggesting a functional heterogeneity within the LC neuron population [165].

**Fig. 2:**
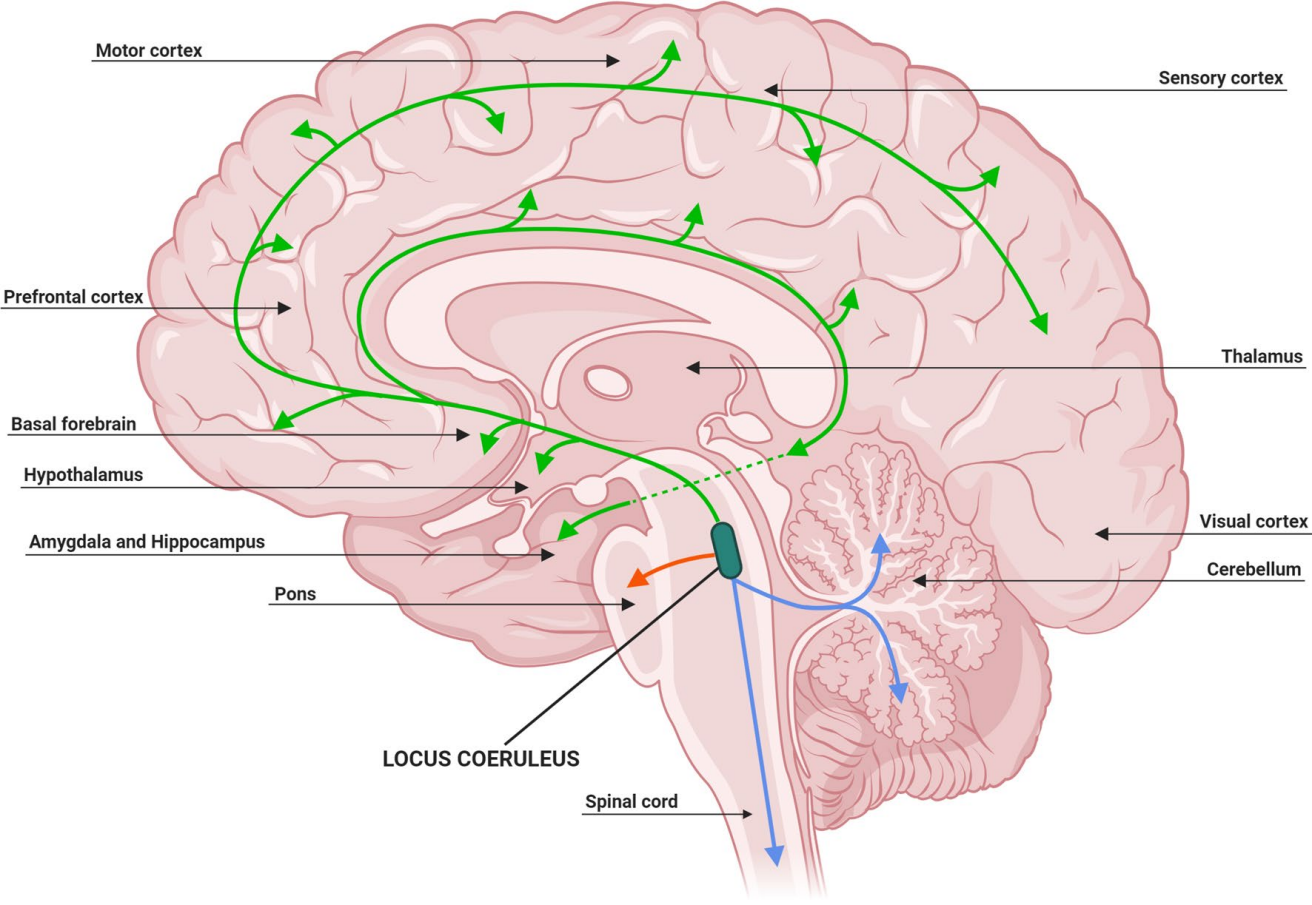
Locus coeruleus projections release norepinephrine throughout the central nervous system. The organization of noradrenergic neurons within the locus coeruleus is indicative of their projection destination. Rostral locus coeruleus neurons supply norepinephrine to the forebrain region (green), middle locus coeruleus neurons to the pons (red) and caudal locus coeruleus neurons project to the spinal cord and cerebellum (blue). Adapted from [37, 41, 94]. Created with BioRender.com

The structure and organization of neurons within the LC and adjacent subcoeruleus (SubC) are essential for its function as the primary source of NE. Retrograde labeling techniques using radioactive or fluorescent tracers have established that each single LC axon innervates extensively into several brain regions, in particular the forebrain, cerebellum, brainstem and spinal cord [122] (Fig. 2), with 90% of these LC efferent projections remaining ipsilateral [95, 128]. Several groups have identified that topographic organization of the LC neurons are dependent on their output target, with rostrally located cells projecting to the forebrain region, innervating the hippocampus and septum; whereas, cells in the middle and caudal portions of the LC project to the cerebellum, basal ganglia and spinal cord, and regulate autonomic function [86, 95, 122, 136] (Fig. 2).

LC neuronal projections to the cortex occur in three ways: (1) monosynaptically, whereby the axons innervate the region directly [38, 112]; (2) through the thalamus, using this structure as a ‘hub’ [47, 76]; or (3) via the cholinergic basal forebrain nuclei, including the nbM [53]. At the projection target area, NE is released by two different types of LC axonal terminals; typical synaptic transmission and varicosities [41, 94, 134]. Axonal varicosities may permit the extra-synaptic release of NE commonly referred to as volume transmission, which refers to the diffuse release of neurotransmitters [41, 94, 134]. This method of release is an important and essential characteristic of neuromodulatory neurons as it influences the excitability and synaptic plasticity of a vast range of proximal cells [41, 94, 134].

Once released, NE exerts its neuromodulatory effects through binding to G-coupled α- and β-adrenoreceptors (Fig. 1), which are present on neurons, glia and immune cells, and can be detected in cerebral microvessels [124, 127, 147]. One of the most important receptors in mediating the downstream effects of NE is the α_2_-adrenoreceptor [57, 127] that functions as both inhibitory autoreceptor on noradrenergic neurons and modulator of noradrenergic innervation on postsynaptic cells [127]. α_2_-Adrenoreceptors are also highly expressed by LC neurons, contributing to the neuromodulatory effects of NE [138]. The α_2_-adrenoreceptor subtype, α_2A_-adrenoreceptor, is found abundantly in the cortex, hippocampus, hypothalamus, amygdala, brainstem, and spinal cord; regions that are highly innervated by LC-projecting axon terminals [78, 87, 127]. The abundance of the α_2A_-adrenoreceptor subtype in such regions suggests that it may play an important role in mitigating NE signaling [78, 87, 127]. On binding to adrenoreceptors, NE initiates a vast array of downstream pathways, leading to further neurotransmitter release, regulation of inflammatory processes and growth factor expression, all of which are important in modulating its many roles in the brain [94, 127].

It is widely recognized that NE plays a critical role in regulating neuroinflammation. For example, LC axonal terminals were found in close contact to astrocytes and microglia [23, 83], suggesting a modulatory relationship. In early in vitro studies, NE was shown to inhibit the induction of MHC class II antigen expression on astrocytes by IFN-γ, indicating that NE downregulates the immune response in the brain [45]. Since then, several studies have shown that NE plays a fundamental role in reducing a number of inflammatory genes, including NOS2, IL-1β, ICAM-1, adhesion molecules, and TNF-α, in cells such as microglia, astrocytes and endothelial cells [27, 40, 99].

Findings indicate that much of the neuromodulatory effects of NE occur through downstream cAMP pathways. Through the induction of this pathway, NE was shown to increase the expression of peroxisome proliferator-activated receptor gamma (PPARγ) in astrocytes and neurons, and research suggests that this upregulation partly mediates the anti-inflammatory effects of NE [74]. This is supported by the findings which suggest that treatment with PPARγ agonists restores the depleted levels of inflammatory markers that are observed in LC-lesioned animal models, as well as inhibiting the activation of proinflammatory molecules such as nuclear factor-κB (NFκB) [59] (Table 1). Another mechanism by which NE exhibits its neuromodulatory effects includes increased IκBα expression, a protein involved in inhibiting the NFκB pathway whose action is highly immunosuppressive [48]. This is supported by findings that animals with LC degeneration have reduced basal levels of inhibitory IκB proteins [59].

**Table 1:** Animal model studies used in locus coeruleus research

References in order of appearance	Model(s)	Species	LC Lesion type	Section(s)
Heneka et al*.* 2003 [59]	Control	Rat	DSP-4	Neuroanatomy and function of the locus coeruleus
Heneka et al*.* 2006 [61]	APP23	Mouse	DSP-4	Locus coeruleus atrophy in Alzheimer’s disease
Kalinin et al*,* 2007 [67]	APP V717F	Mouse	DSP-4	Locus coeruleus atrophy in Alzheimer’s disease
Kelly et al*.* 2019 [72]	Tg344-19	Rat	DBH-sap	Locus coeruleus atrophy in Alzheimer’s disease
Hammerschmidt et al*.* 2013 [56]	APP/PS1	Mouse	DBH^−/−^	Locus coeruleus atrophy in Alzheimer’s disease; l-DOPS (droxidopa)
Chalermpalanupap et al*.* 2018 [19]	Tau P301S	Mouse	DSP-4	Locus coeruleus atrophy in Alzheimer’s disease
Heneka et al*.* 2002 [58]	Control	Rat	DSP-4	Locus coeruleus atrophy in Alzheimer’s disease
Heneka et al*.* 2010 [60]	APP V717I & APP/PS1	Mouse	DSP-4	Locus coeruleus atrophy in Alzheimer’s disease
Kang et al*.* 2020 [69]	Tau P301S & MAPT/AEP^−/−^	Mouse	DBH^−/−^	Neuronal vulnerability in the locus coeruleus
Castren et al*.* 1995 [18]	Control	Rat	None	The role of locus coeruleus in neuroprotection
Fawcett et al*.* 1998 [39]	DBH–BDNF	Mouse	None	The role of locus coeruleus in neuroprotection
Mastsunaga et al*.* 2004 [97]	Control	Rat	None	The role of locus coeruleus in neuroprotection
Nakai et al*.* 2006 [114]	Control	Rat	None	The role of locus coeruleus in neuroprotection
Ádori et al*.* 2015 [1]	SSTR2^−/−^	Mouse	None	The role of somatostatin in neuroprotection
O'Meara et al*.* 2000 [115]	GAL^−/−^	Mouse	None	Neuropeptides expressed by the locus coeruleus exhibit neuroprotective effects
Elliott-Hunt et al*.* 2004 [33]	GAL^−/−^ & GAL overexpression	Mouse	None	Neuropeptides expressed by the locus coeruleus exhibit neuroprotective effects
Kalinin et al*.* 2012 [68]	5xFAD	Mouse	None	l-DOPS (droxidopa)
Devi et al*.* 2012 [28]; Zhang et al*.* 2014 [171]	5xFAD	Mouse	None	7,8-Dihydroxyflavone
Takeda et al*.* 1984 [150]	Control	Mouse	None	Vindeburnol
Labatut et al*.* 1988 [77]	Control	Rat	None	Vindeburnol
Braun et al*.* 2014 [13]	5xFAD	Mouse	None	Vindeburnol
Wang et al*.* 2010 [166]; Sun et al*.* 2012 [144]	3xTgAD	Mouse	None	Allopregnanolone
Torres-Sanchez et al*.* 2018 [158]	Control	Rat	None	Brain stimulation technologies
Follesa et al*.* 2007 [42]	Control	Rat	None	Brain stimulation technologies
Rorabaugh et al*.* 2017 [129]	TgF344-AD	Rat	None	Brain stimulation technologies

*LC *locus coeruleus, *DSP-4 N*-(2-chloroethyl)-*N*-ethyl-bromo-benzylamine, *APP *amyloid precursor protein, *PS1 *presenilin-1, *DBH *dopamine β-hydroxylase, *BDNF *brain-derived neurotrophic factor, *SSTR2 *Somatostatin Receptor 2 gene, *GAL *galanin gene, *MAPT *tau gene, *AEP *Asparagine endopeptidase

In addition to releasing neurotransmitters, the LC also co-transmits a variety of neuropeptides, most namely neuropeptide Y and galanin [79], which are expressed in 20% and 80% of neurons, respectively [62]. Studies have suggested that the co-expression of these neuropeptides helps regulate central adrenergic transmission mediated through α_2_-adrenoreceptors [163]. Furthermore, these cells express essential neurotrophins, including brain-derived neurotrophic factor (BDNF), which dictates neuronal survival and differentiation [18, 25], as well as receptors for neuropeptide Y, galanin, somatostatin and hypocretin/orexin [41].

## AD-related neuropathologic changes in the locus coeruleus

### Hyperphosphorylated tau accumulation in the locus coeruleus

**Fig. 3:**
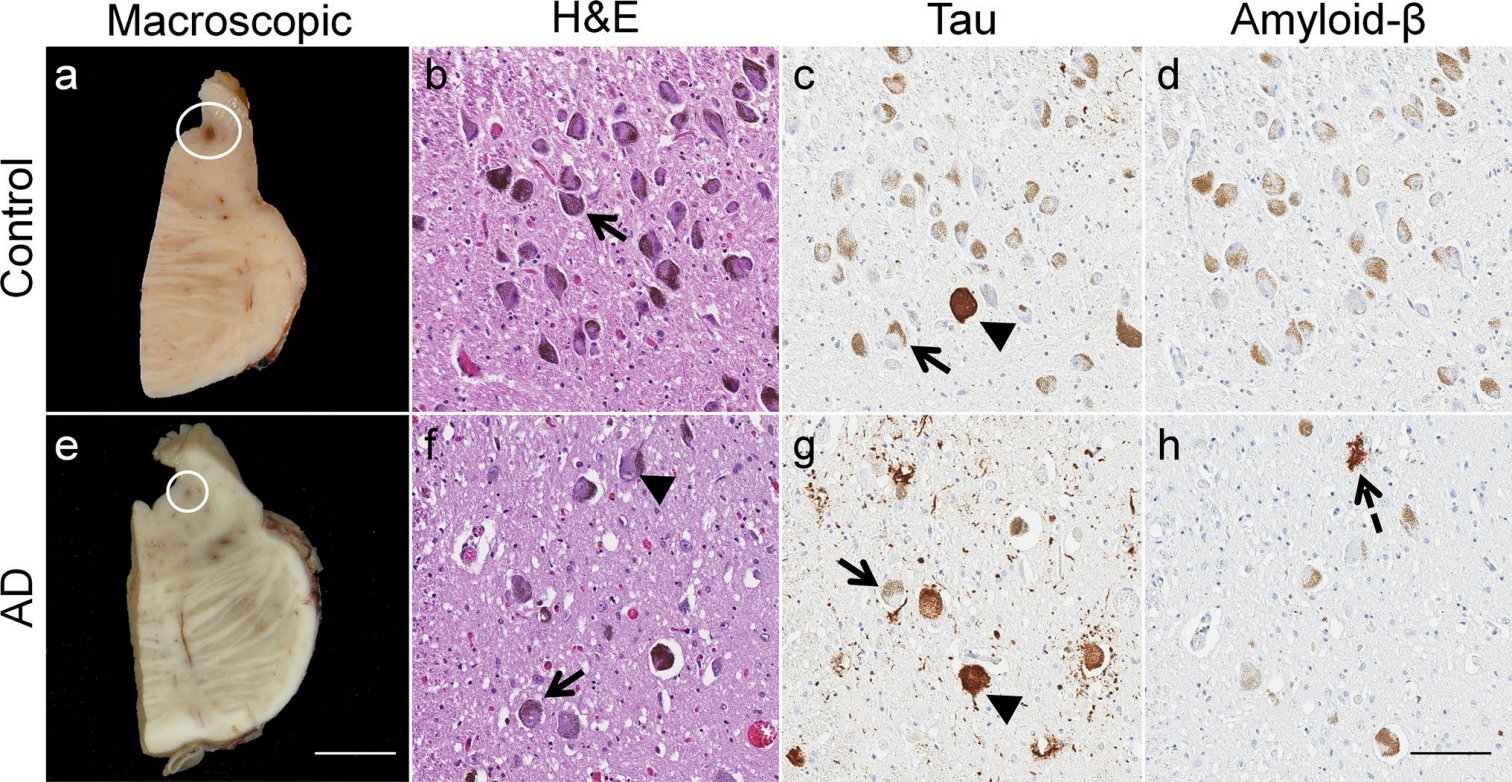
Histopathologic changes in the locus coeruleus during Alzheimer’s disease. **a** Macroscopic examination of the locus coeruleus in an 81-year-old nondemented female revealed a darkly pigmented nucleus due to the presence of neuromelanin (circle). **b** Microscopic inspection of routine hematoxylin and eosin (H&E) stained section demonstrates a well-populated nucleus with neuromelanin-containing neurons (arrow). **c** Isolated neurofibrillary tangles (arrowhead) were identified using tau immunohistochemistry (dark brown), which contrasts with the more yellow–brown appearance of healthy, neuromelanin-containing neurons (arrow). **d** No amyloid deposits were identified in the nondemented control. **e** Macroscopic examination of the brain of an 80-year-old female neuropathologically diagnosed with Alzheimer’s Disease revealed a significantly depigmented locus coeruleus (circle), **f** reflective of the decrease in the population of pigmented neurons (arrow) observed microscopically on an H&E-stained section. Neurofibrillary tangles visibly displace neuromelanin (arrowhead). **g** Tau immunostaining reveals neurofibrillary tangles (arrowhead), which contrast in comparison to the yellowish-brown neuromelanin pigment observed in surviving neurons. **h** An isolated amyloid-β deposit was visible (dotted arrow). Case characteristics—Nondemented control: Braak stage = 0, Thal phase = 0; Sporadic Alzheimer’s disease: Braak stage = V, Thal phase = 5; Immunohistochemistry—Tau marker: PHF-1, Amyloid-β marker: 6F/3D; Scale bar— Macroscopic = 8 mm; Microscopic—100 μm

During the development of AD, NFT pathology progresses in the cortex in six stages: in stages I/II, NFTs are present in the transentorhinal and entorhinal cortex; stages III/IV, NFTs appear in the limbic system and temporal cortices; and finally stages V/VI, where NFTs are present throughout the association and primary cortices [10]. However, a recent revision of the original staging system by Braak and colleagues in 2011 has incorporated the LC as the earliest site of p-tau accumulation [12]. According to the updated staging, ‘pretangle stage a/b’ refers to p-tau accumulation confined only to the pontine tegmentum, in or in close proximity to the LC [12]. In ‘pretangle stage a’, the LC cell processes are positive for AT8 immunoreactivity; whereas in ‘pretangle stage b’, p-tau has extended further down the axon with spiked protrusions along the neuron outer somatic rim [12]. Further research indicated that at Braak stage 0, 8% of LC neurons are p-tau-positive, which doubled by Braak stage I [32]. By Braak stage VI, 100% of LC neurons had tau pathology [5] (Fig. 3).

Recent studies have shown that p-tau begins to accumulate in the LC early in life, in some cases as young as 10 years of age, with up to 90% of individuals having some tau pathology in the LC by the age of thirty [11] (Fig. 3). Similar findings were reported by other groups who observed tau lesions, including NFTs and neuropil threads, in 72% of individuals aged 31–40 years, and 94% of individuals aged 41–50 years [123]. In terms of the onset of AD, it is suggested that NFT formation in the LC occurs 10 years prior to any cognitive changes [20, 55, 153] and at least 25 years before significant neuronal loss [16].

The presence of NFTs in the LC can have a multitude of effects regarding differential gene expression. NFT accumulation in the LC is associated with colocalized expression of tau kinases, including MAPK/ERK, SAPK-JNK, p38, glycogen synthase kinase (GSK) and GSK-3β, with several kinases showing immunoreactivity restricted to neurons bearing p-tau [2]. In addition, NFTs in the LC have increased colocalization with neuroketal adducts (oxidation product of docosahexenoic acid) [2], which was shown to rapidly induce protein cross-linking and aggregation, and are markers of age-related neurodegeneration [29]. Further, NFT accumulation in the LC was shown to correlate with decreased expression of superoxide dismutase 1, suggesting a reduced response to oxidative stress [2]. LC neurons containing truncated tau were shown to have decreased levels of voltage-dependent anion channels, suggesting a decrease in the number of functional mitochondria [2]. Whole-transcriptome analysis of the LC at Braak stage IV identified upregulated expression of proteins associated with heat shock protein-binding and ATP metabolism, and downregulation of DNA-binding and members of the small nucleolar RNAs family protein expression [2]. This suggests that abnormal tau accumulation triggers multiple dynamic changes in LC neurons, perhaps as compensatory mechanisms or to induce cell death. Similarly, active caspase-6 antibody positivity, a marker of apoptosis, was shown to increase in NFT-bearing LC neurons with advancing Braak stage [155]. In contrast, macroautophagy, identified by the autophagosome marker LC3, was found to be decreased during AD progression [155]. As macroautophagy is responsible for the degradation of misfolded proteins and was shown to reduce p-tau accumulation in affected neurons [66], dysregulation of this system could contribute to the formation of NFTs in the LC, ultimately leading to activation of caspase-6 and subsequent neuronal death [155]. Furthermore, neurons associated with aggrecan-containing perineuronal nets are spared from the formation of NFTs [14]. Perineuronal nets are assemblies of extracellular matrix proteins that are implicated in neuroprotection and neural activity regulation [168], thus suggesting a protective mechanism against the development of AD pathology [14]. Interestingly, the LC, which is highly affected by tau pathology, is devoid of perineuronal nets, and its neurons are not contacted by aggrecan-immunoreactive axonal coats, indicating a heightened vulnerability to tau-associated neuronal death [104].

### Amyloid-β in the locus coeruleus

Despite the early implications of p-tau and the LC, Aβ plaques do not occur in this region until Thal amyloid phase 5 [152], where 62% of cases were found to have Aβ deposits (Fig. 3). However, studies by Muresan and Muresan indicated that LC-derived cell lines are prone to intracellular Aβ accumulation in both the neurites [109] and at the axonal terminals [110]. Interestingly, intracellular Aβ accumulation in these cells did not induce apoptosis or necrosis [109]. With projection sites from LC neurons innervating the entire brain, the group hypothesized that early intracellular accumulation of Aβ at axonal terminals could serve as seeds for further Aβ propagation and aggregation [110]. Furthermore, recent findings have indicated that Aβ oligomers may be involved in the abnormal phosphorylation of tau [170]. Aβ oligomers were shown to bind to an allosteric site on α_2A_-adrenoreceptor, which are present on LC neurons, inducing a redirection of NE-induced signaling to GSK-3β [170]. GSK-3β induces tau phosphorylation, and therefore, activation of this pathway by Aβ oligomers may lead to tau hyperphosphorylation and the development of tau-related pathology in AD [170]. This could implicate Aβ oligomers in the susceptibility of noradrenergic neurons of the LC to NFT formation, a hypothesis that needs to be explored in the future.

### Neuronal cell loss

The AD brain was shown to have marked reductions in the size and volume of the LC (Fig. 3). Early studies reported that the rostrocaudal extent of the nucleus shrinks by an average of 6 mm in these patients [49]. More recently, using unbiased stereological analysis, reports have shown that as the Braak stage increases by one unit, the LC volume decreases by 8.4% [154]. Furthermore, 3T magnetic resonance imaging (MRI) assessment of LC signal intensity, which is determined by the intrinsic neuromelanin-driven contrast and, thus, may correlate with LC neuron population [7], showed a marked decrease in the LC signal in AD dementia patients. This finding was regardless of clinical presentation and independent of the [^11^C]-Pittsburgh Compound-B-positron emission tomography (PiB-PET) Aβ load, which correlated with memory performance in typical AD [117]. Although it did not reach significance, AD dementia patients with an atypical (non-amnestic) clinical presentation were observed to have a lower LC signal than typical AD dementia patients [117]. It will be important for future studies to further investigate whether potential differences exist in atypical AD dementia patients; especially given they are more commonly younger at symptom onset [111]. A recent study using MRI to examine brainstem volumetric changes indicated that participants with neuropsychologically confirmed mild cognitive impairment or clinically diagnosed AD dementia had smaller LC volumes as compared to cognitively normal individuals [31].

Reduction in LC volume can be reflected by a vast loss of noradrenergic neurons or neuronal shrinkage. Studies have shown that LC neuronal loss can average 63% in AD [49] and occurs midway through disease progression [154]. Increased neuronal loss is also associated with earlier age of onset [169], increased disease duration, and earlier age of death [8]. In terms of clinical presentation, 30% of LC neurons are lost between progression from cognitively unimpaired to amnestic mild cognitive impairment, with an additional 25% reduction of LC neurons during progression to mild/moderate AD [71]. An unbiased stereological study using neuropsychological and neuropathologic measures to determine AD stage reported that LC neuronal loss is detectable at preclinical stages, and that progressive loss of LC neurons parallels the progression of the disease to severe dementia [3]. Interestingly, Hoogendijk and colleagues reported that AD dementia patients exhibit an 82% and 39% decrease in the number of large multipolar and small fusiform neurons, respectively [64]. This manifested in a reduction in the large to small pigmented neuron ratio, suggesting that decreased LC volume and neuronal count in AD is not entirely due to cell death, but also cell shrinkage and phenotype loss [64]. Similarly, it was reported that LC neurons display characteristic morphological changes in the early stages of AD, such as contracted dendrites and swollen cell bodies, which could also augment noradrenergic dysfunction [153].

It is widely recognized that a vulnerability gradient of the LC across the rostrocaudal axis exists in AD and differs between neurodegenerative diseases. The rostral portion is affected to a greater extent in AD [32], with an 83% loss of length, as compared to the middle and caudal parts (23% and 15%, respectively) [64]. The degree of rostral degeneration was shown to correlate with aggressive behavior in patients with dementia [98]. With rostral neurons projecting to the forebrain and cortical structures, degeneration of this portion could explain behavioral changes observed in AD dementia patients.

To reduce the consequences of LC pathology, many compensatory mechanisms are implemented by the remaining noradrenergic neurons in order to maintain its essential role within the brain. Evidence from postmortem AD and Lewy body disease brain studies with significant LC neuronal loss indicated that the noradrenergic system undergoes major changes that are consistent with compensation [149]. This includes increased tyrosine hydroxylase mRNA expression and NE synthesis, reduced NE uptake at synaptic terminals within the surviving neurons, as well as dendritic sprouting into the peri-LC dendritic zone and axonal sprouting in projection areas [148, 149]. Upregulation of neurotransmitter biosynthesis could explain the increase in NE levels in the cerebrospinal fluid of AD dementia patients with severe LC degeneration, which is also associated with poor cognitive function [35, 63, 153].

## Locus coeruleus atrophy in Alzheimer’s disease

LC neuronal dysfunction in neurodegenerative diseases can have a multitude of effects, both locally and at the projection areas. On a local basis, many cellular changes may augment the dysregulation of the noradrenergic system. Unbiased stereological methods have indicated that LC neurons positive for p-tau have significant reductions in synaptic connectivity, as shown by a loss of approximately 50% of synaptophysin-immunoreactive perineuronal dots [2]. Furthermore, microarray analysis using mRNA from LC neurons displayed a significant reduction in expression of genes associated with neuritic and structural plasticity, as well as mitochondrial respiration and redox gene expression, in patients with amnestic mild cognitive impairment and mild/moderate AD as compared to controls [71]. The downregulation of such genes was found to be associated with cognitive deficits [71]. These findings suggest that LC neurons have a lessened functional capacity, especially in terms of receiving activating inputs, which can ultimately lead to decreased noradrenergic signaling.

Loss of NE concentration throughout the brain was reported by many groups. In AD, NE concentrations in the mid-temporal cortices are decreased by 31% [98] and up to 50% only two years after dementia onset [49]. In addition, AD dementia patients who died before the age of 79 and those with greater loss of LC neurons were found to have a NE deficit in the cingulate cortex [8, 131].

LC and NE network dysfunction can also contribute to the early behavioral alterations seen in preclinical AD, such as sleep disturbances and mood changes. During the awake state, LC neurons are highly active, and consistent fluctuations are observed throughout the day for certain waking behaviors [4]. The LC firing frequency then decreases during slow-wave and non-REM sleep, and is completely diminished by the REM stage and throughout paradoxical sleep [4, 156]. Clinical studies have reported a high frequency of AD dementia patients exhibiting sleep disturbances [159], which is associated with disease severity [102, 130] that could be in part due to loss of LC neurons. In addition to sleep deficits, it is widely recognized that noradrenergic dysfunction can lead to the development of mood disorders [126]. NE upregulation was shown to have antidepressant effects in patients with major depression, and with depression being an early symptom of AD dementia, it is possible that early noradrenergic de-innervation could augment this behavioral phenotype [44, 126, 156, 174].

Diminished signaling to projection areas can result in varied phenotypes. In AD, although α_2_-adrenoreceptor expression is increased in the hippocampus at Braak stage I, its expression is reduced at Braak stage IV [2]. Tyrosine hydroxylase expression also declines with age and disease progression, especially within the hippocampus and amygdala at Braak stages III and IV [2]. A study using APP/PS1 mice crossbred with dopamine β-hydroxylase (–/–) mice, which resulted in a mouse with reduced NE production, showed that depletion of NE impaired long-term synaptic plasticity in the hippocampus, leading to spatial memory deficits [56]. NE deficiency also modified the expression of plasticity-related synaptosomal subunit proteins, such as CAMKII and NMDAR [56]. Similarly, inducing LC degeneration using *N*-(2-chloroethyl)-*N*-ethyl-bromo-benzylamine (DSP-4) in an APP transgenic mouse reduced cerebral glucose metabolism, acetylcholinesterase activity and neuronal integrity, leading to increased memory deficits [61]. LC degeneration also led to increased levels of inducible nitric oxide synthase (iNOS) in the projection areas, resulting in augmented neuronal loss in the frontal cortex and hippocampus [61]. Furthermore, lesioning of the LC in a P301S mouse model of tauopathy resulted in spatial and associative memory deficits, as well as exacerbated hippocampal tau burden and increased lethality, suggesting a direct role of LC degeneration in potentiating cognitive deficits and inducing AD-related pathology and mortality [19].

Research has shown, both in human [8, 92, 157] and animal studies [61, 67], that loss of LC neurons leads to increased Aβ deposition. In human studies, neuronal loss across the rostrocaudal axis of the LC was shown to topographically correspond to the distribution of Aβ plaques within the cerebral cortex of AD brains [92]. Post-mortem analysis have shown that loss of neurons from the rostral, middle and caudal portions of the LC correlates with increased Aβ plaque burden in the frontal cortex, temporal cortex and occipital cortex, respectively [92].

In animal models, DSP-4-treated mice display a fivefold increase in Aβ plaque burden and increased average plaque size [67]. Furthermore, LC-lesioned animals exhibit increased levels of APP C-terminal cleavage fragments, as well as decreased expression and activity of the Aβ-degrading enzyme neprilysin, both of which correlated with augmented Aβ pathology [67]. Similarly, other studies using LC-lesioned rats revealed 30% and 20% increases in the deposition of Aβ in the cortex and hippocampus, respectively [72]. Interestingly, mice with diminished abilities to produce NE do not exhibit exacerbated Aβ deposition, suggesting that LC degeneration itself causes the increase in Aβ levels, and it is probable that such mechanisms are independent of NE signaling, specifically [56].

With the noradrenergic system highly innervating the cerebrovasculature, it is no surprise that LC degeneration can have a major effect on its integrity. Loss of the neural plexus originating from the LC results in increased immunologic infiltration of the brain parenchyma microvasculature and thickening of capillary walls, subsequently leading to the development of microangiopathy and alterations to normal blood–brain barrier functions, a common phenotype of AD [137]. In animal models, lesioning of the LC resulted in vast Aβ deposition within the prefrontal cortex microvessels, indicating the neuropathologic onset of cerebral amyloid angiopathy [72]. In addition, LC-lesioned rats exhibited increased albumin staining, which suggests decreased blood–brain barrier integrity and increased microvasculature permeability and leakage [72]. Moreover, loss of LC–NE signaling also resulted in significant compensatory remodeling of the arterioles in these animals, as displayed by a 45% increase in wall-to-lumen ratio [72]. Together, these findings suggest that LC degeneration plays a key role in the dysregulation of the cerebrovascular system, as seen in AD.

Many studies have suggested that the dysfunction of the noradrenergic system potentiates the neurotoxic proinflammatory condition seen in AD. The presence of NFTs in the LC is associated with the upregulation of inflammatory markers, as well as increased microglia infiltration into the LC [2]. LC-lesioned P301S mice exhibit increased activation of microglia and astrocytes in the hippocampus, which may contribute to hippocampal degeneration in this model [19]. LC cell loss also resulted in amplified microglial and astroglial activation in the frontal cortex and hippocampus in mutant APP mouse models [67] and, thus, the dysregulation of the LC–NE system may play a major role in disease-related inflammation.

Studies have indicated that LC degeneration exacerbates the proinflammatory response to Aβ deposition in the cortex sooner, more robustly, and with longer duration than in control animals [58]. Intriguingly, Aβ injection induced expression of iNOS in microglia in control animals, as compared to LC-lesioned animals whose iNOS expression was localized to neurons, which is in parallel to AD brains [58]. The proinflammatory condition was attenuated by injection of either NE or a β-adrenergic receptor agonist, suggesting that both NE depletion and LC degeneration enhance neuroinflammation and neuronal cell death in AD [58]. Similarly, research shows that NE protects cortical neurons from cytotoxic microglia by decreasing NOS2 production, leading to reduced cell death [88]. Further studies by the same group revealed that LC lesioning leads to increased glial inflammation and Aβ deposition in LC projection areas, including the hippocampus and frontal cortex; whereas, non-projection areas such as the paraventricular thalamus remain unaffected [61]. Findings suggest that increased Aβ deposition following LC degeneration may be in part due to impaired recruitment of microglia to Aβ plaque sites, as well as diminished capabilities of the glial cells to phagocytose Aβ [60]. These findings suggest LC degeneration contributes substantially to the development of AD pathology and disease onset.

## Neuronal vulnerability in the locus coeruleus

The extent to which the LC is affected in AD suggests that its neurons are especially vulnerable to neuropathologic changes [167]. One hypothesis that could confer this susceptibility is the cytoarchitecture of these cells, as LC neurons have long and thin axons that have poor or incomplete myelination [153]. Such a characteristic forces the cell to increase its energy output to perform efficient action potentials that lead to increased cellular oxidative stress, and gaps in the myelin sheath allow greater exposure to environmental toxins [153].

Research has shown that the LC innervates the vast majority of the brain microvasculature, with the estimation that, on average, each LC neuron is responsible for innervating 20 m of capillaries, a coverage that surpasses any other neuron [118]. The exposure of LC neurons to the circulatory system of the brain suggests that they can readily take up environmental toxins from the blood, and the extent to which this occurs can be increased when blood–brain barrier integrity is lost, such as in AD [73, 135]. Furthermore, the LC is in close proximity to the fourth ventricle, which enhances its exposure to the toxins present in the cerebrospinal fluid [107]. Research has shown that the LC is sensitive to the presence of environmental toxins, and increased levels of heavy metals, such as mercury, bismuth and silver, were reported in the LC neurons of AD brains [41, 119]. Studies suggest that accumulation of heavy metals may lead to the development of AD pathology within the LC [141]. Interestingly, AD brains have approximately the same proportion of LC neurons that contain heavy metals (19%) as those that contain p-tau (21%) [119], suggesting that insult to the LC via the accumulation of environmental toxins plays an important role in its degeneration and dysfunction.

Research has suggested that molecular mechanisms may contribute to the development of early tau pathology in selectively vulnerable LC neurons. 3,4-Dihydroxyphenylglycolaldehyde, a metabolic product of NE that is produced exclusively in noradrenergic neurons, was shown to activate asparagine endopeptidase cleavage of tau into aggregation- and propagation-prone forms [69]. 3,4-Dihydroxyphenylglycolaldehyde-induced tau aggregation resulted in LC neurotoxicity, propagation of tau pathology to interconnected brain regions and cognitive impairment in an AD mouse model [69]. Furthermore, activation of asparagine endopeptidase and subsequent tau cleavage required the presence of NE [69], suggesting that production and ultimately the metabolism of NE by noradrenergic neurons could potentiate the selective vulnerability of the LC to neuropathologic changes.

Another factor that may exacerbate the vulnerability of LC neurons is their high bioenergetic need [167]. To conserve their essential physiological function within the brain, LC neurons sustain their spiking rate by exhibiting autonomous activity in the absence of glutamate and γ-aminobutyric acid (GABA) inputs [133]. This is achieved through activity-dependent Ca^2+^ entry, which upon activation, leads to the induction of mitochondrial oxidative stress and results in increased LC neuronal vulnerability [133].

Epidemiological studies have also identified a link between NE availability and an increased risk of developing AD dementia. Polymorphisms in the dopamine β-hydroxylase gene, which influences NE availability in the brain, cause a significant decrease in NE concentration and individuals carrying such polymorphism exhibit a twofold increased risk of developing AD dementia [24].

## The role of locus coeruleus in neuroprotection

NE was shown to have an essential role in neuroprotection by modulating inflammation, stress, and Aβ-mediated neurotoxicity [26, 82, 88, 162]. To exert its neuroprotective effect, NE binds to β_1_- and β_2_-adrenergic receptors to activate downstream signaling pathways involving cAMP response element-binding protein, subsequently leading to the expression of endogenous nerve growth factor (NGF) and BDNF [26]. Interestingly, NGF was found to inhibit tau accumulation and phosphorylation in rat hippocampal neurons by activating cAMP-dependent pathways [172]. Significant loss of NE levels following LC degeneration can, therefore, exacerbate many mechanisms involved in inducing neuronal death and subsequent brain atrophy.

Several studies have shown that many of the neuroprotective mechanisms of NE are implemented by BDNF, which plays a major role in influencing axonal branching, outgrowth and synaptic plasticity [65, 84]. Accumulating evidence suggests that the Trk family of tyrosine protein kinase receptors, namely TrkB, is activated by BDNF to mediate its biological function [6]. This is supported by findings which show that the neuroprotective effects of NE are inhibited by the Trk receptor antagonist K252a [82].

The involvement of the noradrenergic system in the production of BDNF could follow the neurotrophic factor hypothesis, which states that such factors are produced and released by neurons to modulate differentiation and survival of the neurons that they innervate [39]. In animal models, LC neurons were shown to produce a large amount of BDNF [18], which is transported from the cell soma into the noradrenergic projection terminals that widely innervate many regions of the brain [39]. Furthermore, the upregulation of BDNF in noradrenergic neurons leads to increased activation of TrkB throughout the innervated neocortex, which results in long-term cortical morphologic alterations [39]. Interestingly, exogenous BDNF infusion induced a substantial increase in noradrenergic axon density within the frontal cortex of aged rats, which is not recapitulated in young or middle-aged animals, suggesting that BDNF is essential for maintaining noradrenergic cells within the aged brain [97]. Similarly, exogenous BDNF increased multiple-threshold antidromic response in rat LC neurons, indicating that BDNF stimulates remodeling of presynaptic axon terminals, leading to the functional changes seen within the projection areas of the aging rat brain [114].

Despite their high expression in adult rodent LC neurons, it was reported that BDNF and TrkB expression is very low in adult human LC neurons [151]. However, NE-induced expression of BDNF and TrkB, or lack of, may still be of importance in other brain regions innervated by the noradrenergic system. In terms of neurodegenerative disease, mounting evidence has indicated that the expression of BDNF and TrkB is downregulated in the AD brain [46], especially in hippocampal and neocortical areas that are highly innervated by noradrenergic LC neurons [140]. Likewise, TrkB expression is markedly downregulated in the cholinergic neurons of the nbM throughout AD progression, indicating a fundamental function in disease development [52]. Intriguingly, BDNF immunoreactivity was found in Aβ plaques and NFT-bearing neurons [108] and stimulation of the BDNF/TrkB pathway in mouse cells caused significant dephosphorylation of tau [34], suggesting a neuroprotective role in AD.

### Norepinephrine protects against Aβ-induced neurotoxicity

Recent studies support a significant role of NE in preventing Aβ-induced neurotoxicity. NE was shown to protect human neuroteratocarcinoma cultures from Aβ_1-42_ and Aβ_25-35_ toxicity by preventing increases in intracellular reactive oxygen species, mitochondrial membrane depolarization and caspase activation [26]. Similar results were replicated in human SK-N-SH cells [171].

In animal models, exposing primary rat cortical neurons to oligomeric Aβ_1-42_ peptide resulted in cellular damage, which can be moderately reduced by NE co-treatment [89]. NE incubation upregulated expression of γ-glutamylcysteine ligase but downregulated glutathione peroxidase expression, leading to increased levels of neuronal glutathione [89]. As glutathione plays a major role in neuroprotection by reducing oxidative damage [43], increasing glutathione production is a central mechanism by which NE exerts its protective action against Aβ-induced neuronal death.

Other mechanisms by which NE may enact its neuroprotective effects include modulating the expression of certain chemokines by astrocytes. By binding to β-adrenergic receptors and initiating the downstream effects of cAMP signaling, NE induced a significant increase in chemokine CCL2 expression, which is observed at the level of mRNA and protein [90]. Astrocyte-derived CCL2 was found to prevent neuronal damage induced by glutamate, which is found to be in high concentrations within the AD brain, thus indicating that the protective actions of NE are mediated partially through its activation of astrocytic CCL2 expression [90].

Using dopaminergic neurons in vitro to assess the neuroprotective actions of NE, Troadec and colleagues found that low concentrations of the neurotransmitter promoted long-term survival and function of these cells [162]. The protective mechanism employed was similar to the effect induced by a caspase-8 inhibitor, suggesting that NE may rescue dopaminergic neurons from cell death by inhibiting the activation of apoptosis [162]. Furthermore, NE treatment of dopaminergic neurons resulted in a significant decrease in intracellular reactive oxygen species, indicating that NE can exhibit antioxidant attributes, perhaps through its diphenolic structure [162]. Similar results were replicated in cholinergic neurons [160]. These studies provide mechanistic evidence to support the role of LC degeneration in dopaminergic and cholinergic deficits seen in neurodegenerative diseases.

### The role of somatostatin in neuroprotection

LC neurons express high levels of somatostatin receptors, the binding partner of somatostatin [1]. Somatostatin is a regulatory peptide that is widely expressed throughout the brain and exhibits many different functions [36]. Somatostatin immunoreactivity was observed within Aβ plaques [105] and was shown to modulate Aβ metabolism by activating proteolytic degradation [132]. Interestingly, somatostatin is the most consistently reduced neuropeptide in the brain and cerebrospinal fluid of AD dementia patients [15], and somatostatin receptor expression was shown to be downregulated in the frontal and temporal cortices of AD brains [75].

Somatostatin expression was found to be greatly reduced in the temporal cortex of AD brains in association with aberrant clustering and bulging of tyrosine hydroxylase-positive afferents [1]. Decreasing somatostatin receptor 2 mRNA expression in the LC was observed from Braak stages III/IV and throughout the progression of the disease [1]. Following the generation of animal models, somatostatin receptor knockout mice were found to exhibit selective loss of noradrenergic innervation on a global scale [1]. As expression levels of other major LC transcripts, such as tyrosine hydroxylase, dopamine β-hydroxylase and galanin, remain unchanged, these findings suggest that somatostatin receptor loss plays a major role in LC neuronal vulnerability and contributes to dysfunction of the noradrenergic system as a whole.

### Neuropeptides expressed by the locus coeruleus exhibit neuroprotective effects

Galanin is one of the most abundant neuropeptides in the brain, is highly expressed by LC neurons, and was shown to have implications in the development of neurodegenerative diseases. In AD, studies have observed that galanin expression is preserved within the remaining neurons of the LC, suggesting a neuroprotective effect [101]. Furthermore, galanin-containing fibers are found to be in close proximity to cholinergic basal forebrain neurons, and this innervation is enriched in AD brains, which may suggest neurodegenerative compensation [9]. In animal models, loss of galanin function resulted in vast cholinergic degeneration in the medial septum and basal forebrain and cognitive deficits [115]. Galanin overexpression and activation of its receptor using a high-affinity agonist reduced chemically-induced cell death in the hippocampus of transgenic animals [33]. This provides evidence suggestive of a major role of galanin in the neuroprotective effects of the LC.

### Neuromelanin: toxic or protective?

Neuromelanin, a pigment commonly used to identify noradrenergic neurons, slowly accumulates in the LC with age, followed by a significant decrease within individuals aged 60 and older [139]. Intriguingly, research has shown that neuromelanin may play a neuroprotective and/or a neurotoxic role in AD progression [175]. In this ill-fated rescue, the protective mechanism set in place is hypothesized to initiate a cascade of events that ultimately leads to a toxic state that overwhelms the neuron. In a neuroprotective sense, neuromelanin chelates heavy metals such as mercury, cadmium, lead and iron, and diminishes their toxic nature [175]. However, the accumulation of heavy metals in neuromelanin-containing organelles can result in a neurodegenerative response by which the neurons die and release the toxins into the extracellular space, activating microglia and inducing neurotoxic inflammation [175].

## Possible therapy implication

To compensate for the significant loss of NE following LC de-innervation, several studies have demonstrated that certain therapies could partially reverse the dysregulation of the noradrenergic system, thus minimizing the neurodegenerative process [120, 161] (Table 2). Such treatments could be a promising therapy for not only AD, but also other neurodegenerative diseases where loss of NE and LC atrophy is observed, such as Parkinson’s disease and Lewy body dementia.

**Table 2:** Possible therapeutic interventions

References in order of appearance	Therapeutic method	Target	Mechanism of action
Feinstein et al. 2016 [41]; Hammerschmidt et al. 2013 [56]; Heneka et al. 2010 [60]; Kalinin et al. 2012 [68]	l-DOPS (droxidopa)	DOPA decarboxylase	Increase levels of NE
Zhou 2004 [173]; ClinicalTrials.gov, NCT01522404	Atomoxetine	NE transporter	Increase levels of NE at synapse
Devi et al. 2012 [28]; Du and Hill, 2015 [30]; Liu et al. 2010 [81]; Zhang et al. 2014 [171]	7,8-Dihydroxyflavone	TrkB receptor	Increase levels of BDNF
Braun et al. 2014 [13]; Ginovart et al. 1996 [51]; Labatut et al. 1988 [77]; Takeda et al. 1984 [150]; Zyzek et al. 1990 [176]	Vindeburnol	Unknown	Increase NE turnover
Charalampopoulos et al. 2005 [22]; Singh et al. 2012 [142]; Sun et al. 2012 [144]; Wang et al. 2010 [166]	Allopregnanolone	GABA_A_ receptor	Increase levels of NE
Torres-Sanchez et al. 2018 [158]	Deep brain stimulation	Multiple brain regions	Increase NE release
Chang et al. 2018 [21]; Follesa et al. 2007 [42]	Vagus nerve stimulation	Vagus nerve	Increase levels of NE and BDNF
Rorabaugh et al. 2017 [129]	Chemogenetics	LC neurons	Rescue impaired reversal learning

*LC *locus coeruleus, *NE *norepinephrine, *BDNF *brain-derived neurotrophic factor, *GABA *γ-aminobutyric acid

### l-DOPS (droxidopa)

l-3,4-Dihydroxyphenylserine (l-DOPS), a synthetic catecholamine that is a precursor of NE, can be used to directly increase levels of NE [41]. l-DOPS can be administered orally and can readily permeate the blood–brain barrier, where it is converted by carboxylation to NE [54] (Fig. 1) and was shown to effectively treat dopamine β-hydroxylase deficiency and neurogenic orthostatic hypotension [41].

In a 5xFAD transgenic mouse model, co-treatment of l-DOPS and the NE reuptake inhibitor atomoxetine was found to increase NE levels in the brain, as well as improve cognitive functions, reduce neuronal cell death, decrease astrocyte activation and increase BDNF and NGF levels in the cortex and hippocampus [68]. In addition, l-DOPS and atomoxetine co-treatment upregulated the expression of neprilysin, leading to an increase of Aβ degradation and, thus, a reduction in Aβ plaque burden [68]. l-DOPS treatment of NE-depleted APP/PS1 mice also partially rescued spatial memory impairment [56], as well as restored microglial migration and Aβ phagocytosis [60]. Together, these findings suggest that l-DOPS is an encouraging therapeutic method in the treatment of AD.

### Norepinephrine transporter inhibitors

The NE transporter, located in the plasma membrane of noradrenergic neurons, facilitates the removal of excess NE from the synaptic cleft and is, thus, the primary mechanism of noradrenergic signaling inactivation [173]. Targeting the NE transporter, which in turn inhibits the reuptake of NE and increases synaptic NE concentration, was shown to alleviate the symptoms of neuropsychiatric disorders such as depression and attention deficit hyperactivity disorder [173]. Therefore, NE transporter inhibitors such as atomoxetine are routinely used as treatment strategies in these diseases. An ongoing clinical trial (ClinicalTrials.gov Identifier: NCT01522404) is evaluating the efficacy of atomoxetine in participants diagnosed with mild cognitive impairment, to reverse the loss of NE in the brain and slow neurodegeneration. As atomoxetine is already an FDA-approved drug that is safe in the elderly, this is a promising therapeutic measure for treating LC–NE dysregulation in AD.

### 7,8-Dihydroxyflavone

BDNF plays an important role in exhibiting the neuroprotective actions of NE. Therefore, increasing brain levels of BDNF could act as a therapeutic mechanism in AD. When given orally, BDNF is degraded by digestive enzymes [30]. When given systemically, BDNF administration is hindered by its inability to penetrate the blood–brain barrier and its incredibly short half-life [30]. Therefore, there is a need for alternative drugs that can exhibit the same effects as BDNF. Recently, 7,8-Dihydroxyflavone (DHF), a small molecular weight BDNF mimetic, was shown to successfully permeate the blood–brain barrier, as well as displaying heightened stability [30].

DHF was shown to effectively induce TrkB phosphorylation and activation [81] and in a 5xFAD mouse model of AD, administration of DHF for 10 days restored TrkB signaling, reduced Aβ burden, and improved cognitive deficits [28]. Using the same mouse model, chronic administration of DHF was found to activate TrkB signaling and prevent Aβ accumulation, leading to restoration of hippocampal synapse number and synaptic plasticity, as well as amending memory deficits [171]. Further, in vitro research indicated DHF treatment of primary neurons protected them from Aβ-induced cell death, as well as promoting synaptogenesis and dendritic branching [171]. Although more research is needed to translate these results to the clinic due to the difference in BDNF levels between humans and rodents, these findings suggest that DHF could be a possible oral therapeutic measure for treating AD.

### Vindeburnol and allopregnanolone

Research has indicated that a select amount of drugs can selectively increase the number of LC neurons, as well as improve their physiological function.

Vindeburnol (also known as RU24722 and BC-19) treatment in animal models increased NE turnover [150], as well as upregulated tyrosine hydroxylase protein expression and its subsequent activity [77]. In the LC, cell count, volume and tyrosine hydroxylase concentration was increased following administration of vindeburnol [51, 176]. In a 5xFAD AD mouse model, vindeburnol treatment induced neuronal maturation in the LC, restored BDNF levels in the hippocampus, and reduced Aβ deposition throughout the brain [13], suggesting that this treatment influences both the LC itself and its noradrenergic projection sites. Interestingly, in vitro studies indicated that vindeburnol upregulates BDNF expression selectively in astrocytes, suggesting that these cells are the drug’s direct target [13].

Allopregnanolone, a neurosteroid that acts as a positive allosteric modulator of the GABA_A_ receptor, was shown to increase NE and dopamine levels and upregulate the expression of tyrosine hydroxylase in vitro [22]. In addition, although studies of allopregnanolone have not elucidated its effects on LC degeneration, animal studies have shown that it is able to induce neurogenesis [166], in addition to promoting regeneration of dopaminergic neurons in the substantia nigra [144] and restoring hippocampal-dependent learning and cognitive deficits [142, 166].

### Brain stimulation technologies

Pilot studies have suggested that brain stimulation technologies, which are used to modulate cognitive function in neuropsychiatric disorders, could help ameliorate cognitive deficits in AD.

In animal studies, deep brain stimulation was reported to increase NE release in the area of stimulation [158]. Deep brain stimulation of the ventromedial prefrontal cortex was shown to increase tyrosine hydroxylase expression in the LC, as well as increase LC neuron activity [158]. Additionally, vagus nerve stimulation was shown to lessen cognitive deficits in AD dementia patients [21]. In a rat model, acute vagus nerve stimulation was shown to increase NE concentration, as well as increase BDNF expression in the hippocampus and cerebral cortex [42]. As it projects to the LC, it is possible that stimulation of the vagus nerve could directly activate the LC, leading to increased NE at projection sites. In addition, stimulation of the LC using chemogenetic approaches could also be an important therapeutic strategy in AD. In a TgF344-AD rat model, impaired reversal learning was rescued by chemogenetic LC activation [129]. Thus, brain stimulation technologies could be used as essential therapeutic strategies in alleviating the cognitive effects of LC–NE deinnervation in AD.

## Conclusion

In conclusion, research has shown that LC degeneration and subsequent loss of the noradrenergic system play a major role in AD pathogenesis. Lack of noradrenergic innervation leads to a multitude of effects, including neurotoxic inflammation, increased neuropathologic burden and vast neuronal death, especially in the cortical projection areas of the LC. This de-innervation can enhance the progression of cognitive deficits and memory impairment in AD. LC signal intensity, as assessed by MRI, could be used as an early biomarker for AD. Furthermore, the implementation of therapeutics such as l-DOPS and brain stimulation technologies could alleviate the neuropathologic processes and symptoms of this disease. Since the LC-NE system is a key player in modulating neuroprotection and regulating neuroinflammation, and its degeneration plays an essential role in the development of AD, a better understanding of the mechanistic link between LC loss and onset of AD could be vital for the diagnosis and therapeutic efforts against disease progression.

## Abbreviations

Aβ: Amyloid-β
AD: Alzheimer’s disease
BDNF: Brain-derived neurotrophic factor
DHF: 7,8-Dihydroxyflavone
DSP-4: *N*-(2-Chloroethyl)-*N*-ethyl-bromo-benzylamine
GABA: γ-Aminobutyric acid
GSK: Glycogen synthase kinase
iNOS: Inducible nitric oxide synthase
LC: Locus coeruleus
l-DOPS: l-3,4-Dihydroxyphenylserine
MRI: Magnetic resonance imaging
nbM: Nucleus basalis of Meynert
NE: Norepinephrine
NFκB: Nuclear factor-κB
NFT: Neurofibrillary tangle
PiB-PET: C-Pittsburgh Compound-B-positron emission tomography
PPARγ: Peroxisome proliferator-activated receptor gamma
p-tau: Hyperphosphorylated tau
SubC: Subcoeruleus

## References

[1] Ádori C, Glück L, Barde S, Yoshitake T, Kovacs GG, Mulder J, Maglóczky Z, Havas L, Bölcskei K, Mitsios N (2015). Critical role of somatostatin receptor 2 in the vulnerability of the central noradrenergic system: new aspects on Alzheimer’s disease. Acta Neuropathol.

[2] Andrés-Benito P, Fernández-Dueñas V, Carmona M, Escobar LA, Torrejón-Escribano B, Aso E, Ciruela F, Ferrer I (2017). Locus coeruleus at asymptomatic early and middle Braak stages of neurofibrillary tangle pathology. Neuropathol Appl Neurobiol.

[3] Arendt T, Bruckner MK, Morawski M, Jager C, Gertz HJ (2015). Early neurone loss in Alzheimer's disease: cortical or subcortical?. Acta Neuropathol Commun.

[4] Aston-Jones G, Bloom FE (1981). Activity of norepinephrine-containing locus coeruleus neurons in behaving rats anticipates fluctuations in the sleep-waking cycle. J Neurosci.

[5] Attems J, Thomas A, Jellinger K (2012). Correlations between cortical and subcortical tau pathology. Neuropathol Appl Neurobiol.

[6] Barbacid M (1994). The Trk family of neurotrophin receptors. J Neurobiol.

[7] Betts MJ, Kirilina E, Otaduy MCG, Ivanov D, Acosta-Cabronero J, Callaghan MF, Lambert C, Cardenas-Blanco A, Pine K, Passamonti L (2019). Locus coeruleus imaging as a biomarker for noradrenergic dysfunction in neurodegenerative diseases. Brain.

[8] Bondareff W, Mountjoy CQ, Roth M, Rossor MN, Iversen LL, Reynolds GP (1987). Age and histopathologic heterogeneity in Alzheimer's disease: evidence for subtypes. Arch Gen Psychiatry.

[9] Bowser R, Kordower JH, Mufson EJ (1997). A confocal microscopic analysis of galaninergic hyperinnervation of cholinergic basal forebrain neurons in Alzheimer's disease. Brain Pathol.

[10] Braak H, Braak E (1991). Neuropathological stageing of Alzheimer-related changes. Acta Neuropathol.

[11] Braak H, Del Tredici K (2011). The pathological process underlying Alzheimer's disease in individuals under thirty. Acta Neuropathol.

[12] Braak H, Thal DR, Ghebremedhin E, Del Tredici K (2011). Stages of the pathologic process in Alzheimer disease: age categories from 1 to 100 years. J Neuropathol Exp Neurol.

[13] Braun D, Madrigal J, Feinstein D (2014). Noradrenergic regulation of glial activation: molecular mechanisms and therapeutic implications. Curr Neuropharmacol.

[14] Brückner G, Hausen D, Härtig W, Drlicek M, Arendt T, Brauer K (1999). Cortical areas abundant in extracellular matrix chondroitin sulphate proteoglycans are less affected by cytoskeletal changes in Alzheimer's disease. Neuroscience.

[15] Burgos-Ramos E, Hervás-Aguilar A, Aguado-Llera D, Puebla-Jiménez L, Hernández-Pinto AM, Barrios V, Arilla-Ferreiro E (2008). Somatostatin and Alzheimer's disease. Mol Cell Endocrinol.

[16] Busch C, Bohl J, Ohm TG (1997). Spatial, temporal and numeric analysis of Alzheimer changes in the nucleus coeruleus. Neurobiol Aging.

[17] Cadwell CR, Scala F, Li S, Livrizzi G, Shen S, Sandberg R, Jiang X, Tolias AS (2017). Multimodal profiling of single-cell morphology, electrophysiology, and gene expression using Patch-seq. Nat Protoc.

[18] Castren E, Thoenen H, Lindholm D (1995). Brain-derived neurotrophic factor messenger RNA is expressed in the septum, hypothalamus and in adrenergic brain stem nuclei of adult rat brain and is increased by osmotic stimulation in the paraventricular nucleus. Neuroscience.

[19] Chalermpalanupap T, Schroeder JP, Rorabaugh JM, Liles LC, Lah JJ, Levey AI, Weinshenker D (2018). Locus coeruleus ablation exacerbates cognitive deficits, neuropathology, and lethality in P301S tau transgenic mice. J Neurosci.

[20] Chan-Palay V, Asan E (1989). Alterations in catecholamine neurons of the locus coeruleus in senile dementia of the Alzheimer type and in Parkinson's disease with and without dementia and depression. J Comp Neurol.

[21] Chang CH, Lane HY, Lin CH (2018). Brain stimulation in Alzheimer's disease. Front Psychiatry.

[22] Charalampopoulos I, Dermitzaki E, Vardouli L, Tsatsanis C, Stournaras C, Margioris AN, Gravanis A (2005). Dehydroepiandrosterone sulfate and allopregnanolone directly stimulate catecholamine production via induction of tyrosine hydroxylase and secretion by affecting actin polymerization. Endocrinology.

[23] Cohen Z, Molinatti G, Hamel E (1997). Astroglial and vascular interactions of noradrenaline terminals in the rat cerebral cortex. J Cereb Blood Flow Metab.

[24] Combarros O, Warden DR, Hammond N, Cortina-Borja M, Belbin O, Lehmann MG, Wilcock GK, Brown K, Kehoe PG, Barber R (2010). The dopamine β-hydroxylase -1021C/T polymorphism is associated with the risk of Alzheimer's disease in the Epistasis Project. BMC Med Genet.

[25] Conner JM, Lauterborn JC, Yan Q, Gall CM, Varon S (1997). Distribution of brain-derived neurotrophic factor (BDNF) protein and mRNA in the normal adult rat CNS: evidence for anterograde axonal transport. J Neurosci.

[26] Counts SE, Mufson EJ (2010). Noradrenaline activation of neurotrophic pathways protects against neuronal amyloid toxicity. J Neurochem.

[27] Dello Russo C, Boullerne AI, Gavrilyuk V, Feinstein DL (2004). Inhibition of microglial inflammatory responses by norepinephrine: effects on nitric oxide and interleukin-1beta production. J Neuroinflammation.

[28] Devi L, Ohno M (2012). 7,8-dihydroxyflavone, a small-molecule TrkB agonist, reverses memory deficits and BACE1 elevation in a mouse model of alzheimer's disease. Neuropsychopharmacology.

[29] Dominguez M, de Oliveira E, Odena MA, Portero M, Pamplona R, Ferrer I (2016). Redox proteomic profiling of neuroketal-adducted proteins in human brain: regional vulnerability at middle age increases in the elderly. Free Radic Biol Med.

[30] Du X, Hill RA (2015). 7,8-Dihydroxyflavone as a pro-neurotrophic treatment for neurodevelopmental disorders. Neurochem Int.

[31] Dutt S, Li Y, Mather M, Nation DA, Alzheimer's Disease Neuroimaging I (2020). Brainstem volumetric integrity in preclinical and prodromal ALZHEIMER'S disease. J Alzheimers Dis.

[32] Ehrenberg AJ, Nguy AK, Theofilas P, Dunlop S, Suemoto CK, Di Lorenzo Alho AT, Leite RP, Diehl Rodriguez R, Mejia MB, Rüb U (2017). Quantifying the accretion of hyperphosphorylated tau in the locus coeruleus and dorsal raphe nucleus: the pathological building blocks of early Alzheimer's disease. Neuropathol Appl Neurobiol.

[33] Elliott-Hunt CR, Marsh B, Bacon A, Pope R, Vanderplank P, Wynick D (2004). Galanin acts as a neuroprotective factor to the hippocampus. Proc Natl Acad Sci USA.

[34] Elliott E, Atlas R, Lange A, Ginzburg I (2005). Brain-derived neurotrophic factor induces a rapid dephosphorylation of tau protein through a PI-3Kinase signalling mechanism. Eur J Neurosci.

[35] Elrod R, Peskind ER, DiGiacomo L, Brodkin KI, Veith RC, Raskind MA (1997). Effects of Alzheimer's disease severity on cerebrospinal fluid norepinephrine concentration. Am J Psychiatry.

[36] Epelbaum J (1986). Somatostatin in the central nervous system: physiology and pathological modifications. Prog Neurobiol.

[37] Espay AJ, LeWitt PA, Kaufmann H (2014). Norepinephrine deficiency in Parkinson's disease: the case for noradrenergic enhancement. Mov Disord.

[38] Fallon JH, Koziell DA, Moore RY (1978). Catecholamine innervation of the basal forebrain II. Amygdala, suprarhinal cortex and entorhinal cortex. J Comp Neurol.

[39] Fawcett JP, Bamji SX, Causing CG, Aloyz R, Ase AR, Reader TA, McLean JH, Miller FD (1998). Functional evidence that BDNF is an anterograde neuronal trophic factor in the CNS. J Neurosci.

[40] Feinstein DL, Heneka MT, Gavrilyuk V, Dello Russo C, Weinberg G, Galea E (2002). Noradrenergic regulation of inflammatory gene expression in brain. Neurochem Int.

[41] Feinstein DL, Kalinin S, Braun D (2016). Causes, consequences, and cures for neuroinflammation mediated via the locus coeruleus: noradrenergic signaling system. J Neurochem.

[42] Follesa P, Biggio F, Gorini G, Caria S, Talani G, Dazzi L, Puligheddu M, Marrosu F, Biggio G (2007). Vagus nerve stimulation increases norepinephrine concentration and the gene expression of BDNF and bFGF in the rat brain. Brain Res.

[43] Forman HJ, Zhang H, Rinna A (2009). Glutathione: overview of its protective roles, measurement, and biosynthesis. Mol Aspects Med.

[44] Forstl H, Burns A, Levy R, Cairns N (1994). Neuropathological correlates of psychotic phenomena in confirmed Alzheimer's disease. Br J Psychiatry.

[45] Frohman EM, Vayuvegula B, Gupta S, Van Den Noort S (1988). Norepinephrine inhibits γ interferon-induced major histocompatibility class II (Ia) antigen expression on cultured astrocytes via β2-adrenergic signal transduction mechanisms. Proc Natl Acad Sci USA.

[46] Fumagalli F, Racagni G, Riva MA (2006). The expanding role of BDNF: a therapeutic target for Alzheimer's disease?. Pharmacogenomics J.

[47] Garcia-Rill E, Kezunovic N, Hyde J, Simon C, Beck P, Urbano FJ (2013). Coherence and frequency in the reticular activating system (RAS). Sleep Med Rev.

[48] Gavrilyuk V, Horvath P, Weinberg G, Feinstein DL (2001). A 27-bp region of the inducible nitric oxide synthase promoter regulates expression in glial cells. J Neurochem.

[49] German DC, Manaye KF, White CL, Woodward DJ, McIntire DD, Smith WK, Kalaria RN, Mann DMA (1992). Disease-specific patterns of locus coeruleus cell loss. Ann Neurol.

[50] German DC, Walker BS, Manaye K, Smith WK, Woodward DJ, North AJ (1988). The human locus coeruleus: computer reconstruction of cellular distribution. J Neurosci.

[51] Ginovart N, Marcel D, Bezin L, Gagne C, Pujol JF, Weissmann D (1996). Tyrosine hydroxylase expression within Balb/C and C57Black/6 mouse locus coeruleus. II. Quantitative study of the enzyme level. Brain Res.

[52] Ginsberg SD, Che S, Wuu J, Counts SE, Mufson EJ (2006). Down regulation of trk but not p75NTR gene expression in single cholinergic basal forebrain neurons mark the progression of Alzheimer's disease. J Neurochem.

[53] Giorgi FS, Ryskalin L, Ruffoli R, Biagioni F, Limanaqi F, Ferrucci M, Busceti CL, Bonuccelli U, Fornai F (2017). The neuroanatomy of the reticular nucleus locus coeruleus in Alzheimer’s disease. Front Neuroanat.

[54] Goldstein DS (2006). l-Dihydroxyphenylserine (L-DOPS): a norepinephrine prodrug. Cardiovasc Drug Rev.

[55] Grudzien A, Shaw P, Weintraub S, Bigio E, Mash DC, Mesulam MM (2007). Locus coeruleus neurofibrillary degeneration in aging, mild cognitive impairment and early Alzheimer's disease. Neurobiol Aging.

[56] Hammerschmidt T, Kummer MP, Terwel D, Martinez A, Gorji A, Pape HC, Rommelfanger KS, Schroeder JP, Stoll M, Schultze J (2013). Selective loss of noradrenaline exacerbates early cognitive dysfunction and synaptic deficits in APP/PS1 mice. Biol Psychiat.

[57] Hein L, Limbird LE, Eglen RM, Kobilka BK (1999). Gene substitution/knockout to delineate the role of alpha 2-adrenoceptor subtypes in mediating central effects of catecholamines and imidazolines. Ann N Y Acad Sci.

[58] Heneka MT, Galea E, Gavriluyk V, Dumitrescu-Ozimek L, Daeschner JA, O'Banion MK, Weinberg G, Klockgether T, Feinstein DL (2002). Noradrenergic depletion potentiates β-amyloid-induced cortical inflammation: implications for Alzheimer's disease. J Neurosci.

[59] Heneka MT, Gavrilyuk V, Landreth GE, O'Banion MK, Weinberg G, Feinstein DL (2003). Noradrenergic depletion increases inflammatory responses in brain: effects on IκB and HSP70 expression. J Neurochem.

[60] Heneka MT, Nadrigny F, Regen T, Martinez-Hernandez A, Dumitrescu-Ozimek L, Terwel D, Jardanhazi-Kurutz D, Walter J, Kirchhoff F, Hanisch UK (2010). Locus ceruleus controls Alzheimer's disease pathology by modulating microglial functions through norepinephrine. Proc Natl Acad Sci USA.

[61] Heneka MT, Ramanathan M, Jacobs AH, Dumitrescu-Ozimek L, Bilkei-Gorzo A, Debeir T, Sastre M, Galldiks N, Zimmer A, Hoehn M (2006). Locus ceruleus degeneration promotes Alzheimer pathogenesis in amyloid precursor protein 23 transgenic mice. J Neurosci.

[62] Holets VR, Hökfelt T, Rökaeus Å, Terenius L, Goldstein M (1988). Locus coeruleus neurons in the rat containing neuropeptide Y, tyrosine hydroxylase or galanin and their efferent projections to the spinal cord, cerebral cortex and hypothalamus. Neuroscience.

[63] Hoogendijk WJ, Feenstra MG, Botterblom MH, Gilhuis J, Sommer IE, Kamphorst W, Eikelenboom P, Swaab DF (1999). Increased activity of surviving locus ceruleus neurons in Alzheimer's disease. Ann Neurol.

[64] Hoogendijk WJG, Pool CW, Troost D, Van Zwieten E, Swaab DF (1995). Image analyser-assisted morphometry of the locus coeruleus in alzheimer's disease, parkinson's disease and amyotrophic lateral sclerosis. Brain.

[65] Inoue A, Sanes JR (1997). Lamina-specific connectivity in the brain: regulation by N-cadherin, neurotrophins, and glycoconjugates. Science.

[66] Inoue K, Rispoli J, Kaphzan H, Klann E, Chen EI, Kim J, Komatsu M, Abeliovich A (2012). Macroautophagy deficiency mediates age-dependent neurodegeneration through a phospho-tau pathway. Mol Neurodegen.

[67] Kalinin S, Gavrilyuk V, Polak PE, Vasser R, Zhao J, Heneka MT, Feinstein DL (2007). Noradrenaline deficiency in brain increases β-amyloid plaque burden in an animal model of Alzheimer's disease. Neurobiol Aging.

[68] Kalinin S, Polak PE, Lin SX, Sakharkar AJ, Pandey SC, Feinstein DL (2012). The noradrenaline precursor L-DOPS reduces pathology in a mouse model of Alzheimer's disease. Neurobiol Aging.

[69] Kang SS, Liu X, Ahn EH, Xiang J, Manfredsson FP, Yang X, Luo HR, Liles LC, Weinshenker D, Ye K (2020). Norepinephrine metabolite DOPEGAL activates AEP and pathological Tau aggregation in locus coeruleus. J Clin Invest.

[70] Kaufmann H, Norcliffe-Kaufmann L, Palma JA (2015). Droxidopa in neurogenic orthostatic hypotension. Expert Rev Cardiovasc Ther.

[71] Kelly SC, He B, Perez SE, Ginsberg SD, Mufson EJ, Counts SE (2017). Locus coeruleus cellular and molecular pathology during the progression of Alzheimer's disease. Acta Neuropathol Commun.

[72] Kelly SC, McKay EC, Beck JS, Collier TJ, Dorrance AM, Counts SE (2019). Locus coeruleus degeneration induces forebrain vascular pathology in a transgenic rat model of Alzheimer's disease. J Alzheimer's Dis.

[73] Kisler K, Nelson AR, Montagne A, Zlokovic BV (2017). Cerebral blood flow regulation and neurovascular dysfunction in Alzheimer disease. Nat Rev Neurosci.

[74] Klotz L, Sastre M, Kreutz A, Gavrilyuk V, Klockgether T, Feinstein DL, Heneka MT (2003). Noradrenaline induces expression of peroxisome proliferator activated receptor gamma (PPARγ) in murine primary astrocytes and neurons. J Neurochem.

[75] Krantic S, Robitaille Y, Quirion R (1992). Deficits in the somatostatin SS1 receptor sub-type in frontal and temporal cortices in Alzheimer's disease. Brain Res.

[76] Krout KE, Kawano J, Mettenleiter TC, Loewy AD (2002). CNS inputs to the suprachiasmatic nucleus of the rat. Neuroscience.

[77] Labatut R, Richard F, Milne B, Quintin L, Lecestre D, Pujol JFF (1988). Long-term effects of RU24722 on tyrosine hydroxylase of the rat brain. J Neurochem.

[78] Lahdesmaki J, Sallinen J, MacDonald E, Kobilka BK, Fagerholm V, Scheinin M (2002). Behavioral and neurochemical characterization of alpha(2A)-adrenergic receptor knockout mice. Neuroscience.

[79] Le Maitre E, Barde SS, Palkovits M, Diaz-Heijtz R, Hokfelt TG (2013). Distinct features of neurotransmitter systems in the human brain with focus on the galanin system in locus coeruleus and dorsal raphe. Proc Natl Acad Sci USA.

[80] Lidbrink P (1974). The effect of lesions of ascending noradrenaline pathways on sleep and waking in the rat. Brain Res.

[81] Liu X, Chan CB, Jang SW, Pradoldej S, Huang J, He K, Phun LH, France S, Xiao G, Jia Y (2010). A synthetic 7,8-dihydroxyflavone derivative promotes neurogenesis and exhibits potent antidepressant effect. J Med Chem.

[82] Liu X, Ye K, Weinshenker D (2015). Norepinephrine protects against amyloid-β toxicity via TrkB. J Alzheimer's Dis.

[83] Liu YU, Ying Y, Li Y, Eyo UB, Chen T, Zheng J, Umpierre AD, Zhu J, Bosco DB, Dong H (2019). Neuronal network activity controls microglial process surveillance in awake mice via norepinephrine signaling. Nat Neurosci.

[84] Lohof AM, Ip NY, Poo MM (1993). Potentiation of developing neuromuscular synapses by the neurotrophins NT-3 and BDNF. Nature.

[85] Lohr JB, Jeste DV (1988). Locus ceruleus morphometry in aging and schizophrenia. Acta Psychiatr Scand.

[86] Loughlin SE, Foote SL, Grzanna R (1986). Efferent projections of nucleus locus coeruleus: morphologic subpopulations have different efferent targets. Neuroscience.

[87] MacDonald E, Kobilka BK, Scheinin M (1997). Gene targeting–homing in on alpha 2-adrenoceptor-subtype function. Trends Pharmacol Sci.

[88] Madrigal JLM, Feinstein DL, Russo CD (2005). Norepinephrine protects cortical neurons against microglial-induced cell death. J Neurosci Res.

[89] Madrigal JLM, Kalinin S, Richardson JC, Feinstein DL (2007). Neuroprotective actions of noradrenaline: effects on glutathione synthesis and activation of peroxisome proliferator activated receptor delta. J Neurochem.

[90] Madrigal JLM, Leza JC, Polak P, Kalinin S, Feinstein DL (2009). Astrocyte-derived MCP-1 mediates neuroprotective effects of noradrenaline. J Neurosci.

[91] Mann DMA (1983). The locus coeruleus and its possible role in ageing and degenerative disease of the human central nervous system. Mech Ageing Dev.

[92] Marcyniuk B, Mann DM, Yates PO (1986). Loss of nerve cells from locus coeruleus in Alzheimer's disease is topographically arranged. Neurosci Lett.

[93] Marcyniuk B, Mann DM, Yates PO (1989). The topography of nerve cell loss from the locus caeruleus in elderly persons. Neurobiol Aging.

[94] Marien MR, Colpaert FC, Rosenquist AC (2004). Noradrenergic mechanisms in neurodegenerative diseases: a theory. Brain Res Brain Res Rev.

[95] Mason ST, Fibiger HC (1979). Regional topography within noradrenergic locus coeruleus as revealed by retrograde transport of horseradish peroxidase. J Comp Neurol.

[96] Mather M, Harley CW (2016). The locus coeruleus: essential for maintaining cognitive function and the aging brain. Trends Cogn Sci.

[97] Matsunaga W, Shirokawa T, Isobe K (2004). BDNF is necessary for maintenance of noradrenergic innervations in the aged rat brain. Neurobiol Aging.

[98] Matthews KL, Chen CPL, Esiri MM, Keene J, Minger SL, Francis PT (2002). Cognition in patients with dementia. Science.

[99] McNamee EN, Griffin EW, Ryan KM, Ryan KJ, Heffernan S, Harkin A, Connor TJ (2010). Noradrenaline acting at beta-adrenoceptors induces expression of IL-1beta and its negative regulators IL-1ra and IL-1RII, and drives an overall anti-inflammatory phenotype in rat cortex. Neuropharmacology.

[100] Mesulam MM (2013). Cholinergic circuitry of the human nucleus basalis and its fate in Alzheimer's disease. J Comp Neurol.

[101] Miller MA, Kolb PE, Leverenz JB, Peskind ER, Raskind MA (2002). Preservation of noradrenergic neurons in the locus ceruleus that coexpress Galanin mRNA in Alzheimer's disease. J Neurochem.

[102] Mirmiran M, Swaab DF, Kok JH, Hofman MA, Witting W, Van Gool WA (1992). Circadian rhythms and the suprachiasmatic nucleus in perinatal development, aging and Alzheimer's disease. Prog Brain Res.

[103] Montine TJ, Phelps CH, Beach TG, Bigio EH, Cairns NJ, Dickson DW, Duyckaerts C, Frosch MP, Masliah E, Mirra SS (2012). National institute on aging-Alzheimer's association guidelines for the neuropathologic assessment of Alzheimer's disease: a practical approach. Acta Neuropathol.

[104] Morawski M, Brückner G, Jäger C, Seeger G, Arendt T (2010). Neurons associated with aggrecan-based perineuronal nets are protected against tau pathology in subcortical regions in Alzheimer's disease. Neuroscience.

[105] Morrison JH, Rogers J, Scherr S, Benoit R, Bloom FE (1985). Somatostatin immunoreactivity in neuritic plaques of Alzheimer's patients. Nature.

[106] Mouton PR, Pakkenberg B, Gundersen HJ, Price DL (1994). Absolute number and size of pigmented locus coeruleus neurons in young and aged individuals. J Chem Neuroanat.

[107] Mravec B, Lejavova K, Cubinkova V (2014). Locus (Coeruleus) minoris resistentiae in pathogenesis of Alzheimer’s disease. Curr Alzheimer Res.

[108] Murer MG, Boissiere F, Yan Q, Hunot S, Villares J, Faucheux B, Agid T, Hirsh E, Raisman-Vozari R (1999). An immunohistochemical study of the distribution of brain-derived neurotrophic factor in the adult human brain, with particular reference to Alzheimer's disease. Neuroscience.

[109] Muresan Z, Muresan V (2006). Neuritic deposits of amyloid-beta peptide in a subpopulation of central nervous system-derived neuronal cells. Mol Cell Biol.

[110] Muresan Z, Muresan V (2008). Seeding neuritic plaques from the distance: a possible role for brainstem neurons in the development of Alzheimer's disease pathology. Neurodegener Dis.

[111] Murray ME, Graff-Radford NR, Ross OA, Petersen RC, Duara R, Dickson DW (2011). Neuropathologically defined subtypes of Alzheimer's disease with distinct clinical characteristics: a retrospective study. Lancet Neurol.

[112] Nagai T, Satoh K, Imamoto K, Maeda T (1981). Divergent projections of catecholamine neurons of the locus coeruleus as revealed by fluorescent retrograde double labeling technique. Neurosci Lett.

[113] Nagatsu T, Levitt M, Udenfriend S (1964). Tyrosine hydroxylase. the initial step in norepinephrine biosynthesis. J Biol Chem.

[114] Nakai S, Matsunaga W, Ishida Y, Isobe KI, Shirokawa T (2006). Effects of BDNF infusion on the axon terminals of locus coeruleus neurons of aging rats. Neurosci Res.

[115] O'Meara G, Coumis U, Ma SY, Kehr J, Mahoney S, Bacon A, Allen SJ, Holmes F, Kahl U, Wang FH (2000). Galanin regulates the postnatal survival of a subset of basal forebrain cholinergic neurons. Proc Natl Acad Sci USA.

[116] Ohm TG, Busch C, Bohl J (1997). Unbiased estimation of neuronal numbers in the human nucleus coeruleus during aging. Neurobiol Aging.

[117] Olivieri P, Lagarde J, Lehericy S, Valabrègue R, Michel A, Macé P, Caillé F, Gervais P, Bottlaender M, Sarazin M (2019). Early alteration of the locus coeruleus in phenotypic variants of Alzheimer’s disease. Ann Clin Transl Neurol.

[118] Pamphlett R (2014). Uptake of environmental toxicants by the locus ceruleus: a potential trigger for neurodegenerative, demyelinating and psychiatric disorders. Med Hypotheses.

[119] Pamphlett R, Kum Jew S (2015). Different populations of human locus ceruleus neurons contain heavy metals or hyperphosphorylated tau: implications for amyloid-β and tau pathology in Alzheimer's disease. J Alzheimer's Dis.

[120] Phillips C, Fahimi A, Das D, Mojabi FS, Ponnusamy R, Salehi A (2016). Noradrenergic system in down syndrome and Alzheimer's disease a target for therapy. Curr Alzheimer Res.

[121] Pickel VM, Joh TH, Reis DJ (1975). Ultrastructural localization of tyrosine hydroxylase in noradrenergic neurons of brain. Proc Natl Acad Sci USA.

[122] Pickel VM, Segal M, Bloom FE (1974). A radioautographic study of the efferent pathways of the nucleus locus coeruleus. J Comp Neurol.

[123] Pletnikova O, Kageyama Y, Rudow G, LaClair KD, Albert M, Crain BJ, Tian J, Fowler D, Troncoso JC (2018). The spectrum of preclinical Alzheimer's disease pathology and its modulation by ApoE genotype. Neurobiol Aging.

[124] Poe GR, Foote S, Eschenko O, Johansen JP, Bouret S, Aston-Jones G, Harley CW, Manahan-Vaughan D, Weinshenker D, Valentino R (2020). Locus coeruleus: a new look at the blue spot. Nat Rev Neurosci.

[125] Ramón-Moliner E, Nauta WJH (1966). The isodendritic core of the brain stem. J Comp Neurol.

[126] Ressler KJ, Nemeroff CB (1999). Role of norepinephrine in the pathophysiology and treatment of mood disorders. Biol Psychiatry.

[127] Rommelfanger KS, Weinshenker D (2007). Norepinephrine: The redheaded stepchild of Parkinson's disease. Biochem Pharmacol.

[128] Room P, Postema F, Korf J (1981). Divergent axon collaterals of rat locus coeruleus neurons: demonstration by a fluorescent double labeling technique. Brain Res.

[129] Rorabaugh JM, Chalermpalanupap T, Botz-Zapp CA, Fu VM, Lembeck NA, Cohen RM, Weinshenker D (2017). Chemogenetic locus coeruleus activation restores reversal learning in a rat model of Alzheimer's disease. Brain.

[130] Ross JA, McGonigle P, Van Bockstaele EJ (2015). Locus coeruleus, norepinephrine and Abeta peptides in Alzheimer's disease. Neurobiol Stress.

[131] Rossor MN, Iversen LL, Reynolds GP, Mountjoy CQ, Roth M (1984). Neurochemical characteristics of early and late onset types of Alzheimer's disease. BMJ.

[132] Saito T, Iwata N, Tsubuki S, Takaki Y, Takano J, Huang SM, Suemoto T, Higuchi M, Saido TC (2005). Somatostatin regulates brain amyloid β peptide Aβ42 through modulation of proteolytic degradation. Nat Med.

[133] Sanchez-Padilla J, Guzman JN, Ilijic E, Kondapalli J, Galtieri DJ, Yang B, Schieber S, Oertel W, Wokosin D, Schumacker PT (2014). Mitochondrial oxidant stress in locus coeruleus is regulated by activity and nitric oxide synthase. Nat Neurosci.

[134] Sara SJ (2009). The locus coeruleus and noradrenergic modulation of cognition. Nat Rev Neurosci.

[135] Satoh A, Iijima KM (2019). Roles of tau pathology in the locus coeruleus (LC) in age-associated pathophysiology and Alzheimer's disease pathogenesis: potential strategies to protect the LC against aging. Brain Res.

[136] Satoh K, Tohyama M, Yamamoto K, Sakumoto T, Shimizu N (1977). Noradrenaline innervation of the spinal cord studied by the horseradish peroxidase method combined with monoamine oxidase staining. Exp Brain Res.

[137] Scheibel AB, Duong TH, Tomiyasu U (1987). Denervation microangiopathy in senile dementia, Alzheimer type. Alzheimer Dis Assoc Disord.

[138] Schwarz LA, Luo L (2015). Organization of the locus coeruleus-norepinephrine system. Curr Biol.

[139] Shibata E, Sasaki M, Tohyama K, Kanbara Y, Otsuka K, Ehara S, Sakai A (2006). Age-related changes in locus ceruleus on neuromelanin magnetic resonance imaging at 3 Tesla. Magn Reson Med Sci.

[140] Siegel GJ, Chauhan NB (2000). Neurotrophic factors in Alzheimer's and Parkinson's disease brain. Brain Res Brain Res Rev.

[141] Simic G, Babic Leko M, Wray S, Harrington CR, Delalle I, Jovanov-Milosevic N, Bazadona D, Buee L, de Silva R, Di Giovanni G (2017). Monoaminergic neuropathology in Alzheimer's disease. Prog Neurobiol.

[142] Singh C, Liu L, Wang JM, Irwin RW, Yao J, Chen S, Henry S, Thompson RF, Brinton RD (2012). Allopregnanolone restores hippocampal-dependent learning and memory and neural progenitor survival in aging 3xTgAD and nonTg mice. Neurobiol Aging.

[143] Stratmann K, Heinsen H, Korf HW, Del Turco D, Ghebremedhin E, Seidel K, Bouzrou M, Grinberg LT, Bohl J, Wharton SB (2016). Precortical phase of Alzheimer's disease (AD)-related tau cytoskeletal pathology. Brain Pathol.

[144] Sun C, Ou X, Farley JM, Stockmeier C, Bigler S, Brinton RD, Wang JM (2012). Allopregnanolone increases the number of dopaminergic neurons in substantia nigra of a triple transgenic mouse model of Alzheimer's disease. Curr Alzheimer Res.

[145] Swanson LW (1976). The locus coeruleus: a cytoarchitectonic, golgi and immunohistochemical study in the albino rat. Brain Res.

[146] Swanson LW, Hartman BK (1975). The central adrenergic system. An immunofluorescence study of the location of cell bodies and their efferent connections in the rat utilizing dopamine-B-hydroxylase as a marker. J Comp Neurol.

[147] Szabadi E (2013). Functional neuroanatomy of the central noradrenergic system. J Psychopharmacol.

[148] Szot P, Leverenz JB, Peskind ER, Kiyasu E, Rohde K, Miller MA, Raskind MA (2000). Tyrosine hydroxylase and norepinephrine transporter mRNA expression in the locus coeruleus in Alzheimer’s disease. Mol Brain Res.

[149] Szot P, White SS, Lynne Greenup J, Leverenz JB, Peskind ER, Raskind MA (2006). Compensatory changes in the noradrenergic nervous system in the locus ceruleus and hippocampus of postmortem subjects with Alzheimer's disease and dementia with lewy bodies. J Neurosci.

[150] Takeda N, Yamatodani A, Watanabe T, Wada H (1984). Effect of a new eburnamine derivative, RU 24722, on the turnover of monoamines in mouse brain: selective and reversible decrease of noradrenaline. Eur J Pharmacol.

[151] Tang S, Machaalani R, Waters KA (2010). Immunolocalization of pro- and mature-brain derived neurotrophic factor (BDNF) and receptor TrkB in the human brainstem and hippocampus. Brain Res.

[152] Thal DR, Rüb U, Orantes M, Braak H (2002). Phases of Aβ-deposition in the human brain and its relevance for the development of AD. Neurology.

[153] Theofilas P, Dunlop S, Heinsen H, Grinberg LT (2015). Turning on the light within: Subcortical nuclei of the isodentritic core and their role in Alzheimer's disease pathogenesis. J Alzheimer's Dis.

[154] Theofilas P, Ehrenberg AJ, Dunlop S, Di Lorenzo Alho AT, Nguy A, Leite REP, Rodriguez RD, Mejia MB, Suemoto CK, Ferretti-Rebustini REDL (2017). Locus coeruleus volume and cell population changes during Alzheimer's disease progression: a stereological study in human postmortem brains with potential implication for early-stage biomarker discovery. Alzheimer's Dementia.

[155] Theofilas P, Ehrenberg AJ, Nguy A, Thackrey JM, Dunlop S, Mejia MB, Alho AT, Paraizo Leite RE, Rodriguez RD, Suemoto CK (2018). Probing the correlation of neuronal loss, neurofibrillary tangles, and cell death markers across the Alzheimer's disease Braak stages: a quantitative study in humans. Neurobiol Aging.

[156] Theofilas P, Heinsen H, Grinberg LT (2016) Brainstem Circuitry and Emotions. Elsevier Inc., City, pp 317–326

[157] Tomlinson BE, Irving D, Blessed G (1981). Cell loss in the locus coeruleus in senile dementia of Alzheimer type. J Neurol Sci.

[158] Torres-Sanchez S, Perez-Caballero L, Mico JA, Celada P, Berrocoso E (2018). Effect of deep brain stimulation of the ventromedial prefrontal cortex on the noradrenergic system in rats. Brain Stimul.

[159] Tractenberg RE, Singer CM, Cummings JL, Thal LJ (2003). The sleep disorders inventory: an instrument for studies of sleep disturbance in persons with Alzheimer's disease. J Sleep Res.

[160] Traver S, Salthun-Lassalle B, Marien M, Hirsch EC, Colpaert F, Michel PP (2005). The neurotransmitter noradrenaline rescues septal cholinergic neurons in culture from degeneration caused by low-level oxidative stress. Mol Pharmacol.

[161] Trillo L, Das D, Hsieh W, Medina B, Moghadam S, Lin B, Dang V, Sanchez MM, De Miguel Z, Ashford JW (2013). Ascending monoaminergic systems alterations in Alzheimer's disease. translating basic science into clinical care. Neurosci Biobehav Rev.

[162] Troadec JD, Marien M, Darios F, Hartmann A, Ruberg M, Colpaert F, Michel PP (2001). Noradrenaline provides long-term protection to dopaminergic neurons by reducing oxidative stress. J Neurochem.

[163] Tsuda K, Yokoo H, Goldstein M (1989). Neuropeptide Y and galanin in norepinephrine release in hypothalamic slices. Hypertension.

[164] Vijayashankar N, Brody H (1979). A quantitative study of the pigmented neurons in the nuclei locus coeruleus and subcoeruleus in man as related to aging. J Neuropathol Exp Neurol.

[165] Wagner-Altendorf TA, Fischer B, Roeper J (2019). Axonal projection-specific differences in somatodendritic alpha2 autoreceptor function in locus coeruleus neurons. Eur J Neurosci.

[166] Wang JM, Singh C, Liu L, Irwin RW, Chen S, Chung EJ, Thompson RF, Brinton RD (2010). Allopregnanolone reverses neurogenic and cognitive deficits in mouse model of Alzheimer's disease. Proc Natl Acad Sci USA.

[167] Weinshenker D (2018). Long road to ruin: noradrenergic dysfunction in neurodegenerative disease. Trends Neurosci.

[168] Wen TH, Binder DK, Ethell IM, Razak KA (2018). The perineuronal 'Safety' net? perineuronal net abnormalities in neurological disorders. Front Mol Neurosci.

[169] Zarow C, Lyness SA, Mortimer JA, Chui HC (2003). Neuronal loss is greater in the locus coeruleus than nucleus basalis and substantia nigra in Alzheimer and Parkinson diseases. Arch Neurol.

[170] Zhang F, Gannon M, Chen Y, Yan S, Zhang S, Feng W, Tao J, Sha B, Liu Z, Saito T (2020). β-amyloid redirects norepinephrine signaling to activate the pathogenic GSK3β/tau cascade. Sci Transl Med.

[171] Zhang Z, Liu X, Schroeder JP, Chan CB, Song M, Yu SP, Weinshenker D, Ye K (2014). 7,8-dihydroxyflavone prevents synaptic loss and memory deficits in a mouse model of Alzheimer's disease. Neuropsychopharmacology.

[172] Zhang ZH, Xi GM, Li WC, Ling HY, Qu P, Fang XB (2010). Cyclic-AMP response element binding protein and tau are involved in the neuroprotective mechanisms of nerve growth factor during focal cerebral ischemia/reperfusion in rats. J Clin Neuroscie.

[173] Zhou J (2004). Norepinephrine transporter inhibitors and their therapeutic potential. Drugs Future.

[174] Zubenko GS, Moossy J, Kopp U (1990). Neurochemical correlates of major depression in primary dementia. Arch Neurol.

[175] Zucca FA, Segura-Aguilar J, Ferrari E, Munoz P, Paris I, Sulzer D, Sarna T, Casella L, Zecca L (2017). Interactions of iron, dopamine and neuromelanin pathways in brain aging and Parkinson's disease. Prog Neurobiol.

[176] Zyzek E, Richard F, Bouilloux JPP, Pujol JFF (1990). Ontogeny of tyrosine hydroxylase concentration in locus coeruleus of newborn rats: long-term effects of RU24722. J Neurochem.

